# Diversity and Spatial Structure of Belowground Plant–Fungal Symbiosis in a Mixed Subtropical Forest of Ectomycorrhizal and Arbuscular Mycorrhizal Plants

**DOI:** 10.1371/journal.pone.0086566

**Published:** 2014-01-28

**Authors:** Hirokazu Toju, Hirotoshi Sato, Akifumi S. Tanabe

**Affiliations:** 1 Graduate School of Global Environmental Studies, Kyoto University, Sakyo, Kyoto, Japan; 2 Graduate School of Human and Environmental Studies, Kyoto University, Sakyo, Kyoto, Japan; 3 National Research Institute of Fisheries Science, Fisheries Research Agency, Fukuura, Kanazawa, Yokohama, Kanagawa, Japan; Institute for Plant Protection (IPP), CNR, Italy

## Abstract

Plant–mycorrhizal fungal interactions are ubiquitous in forest ecosystems. While ectomycorrhizal plants and their fungi generally dominate temperate forests, arbuscular mycorrhizal symbiosis is common in the tropics. In subtropical regions, however, ectomycorrhizal and arbuscular mycorrhizal plants co-occur at comparable abundances in single forests, presumably generating complex community structures of root-associated fungi. To reveal root-associated fungal community structure in a mixed forest of ectomycorrhizal and arbuscular mycorrhizal plants, we conducted a massively-parallel pyrosequencing analysis, targeting fungi in the roots of 36 plant species that co-occur in a subtropical forest. In total, 580 fungal operational taxonomic units were detected, of which 132 and 58 were probably ectomycorrhizal and arbuscular mycorrhizal, respectively. As expected, the composition of fungal symbionts differed between fagaceous (ectomycorrhizal) and non-fagaceous (possibly arbuscular mycorrhizal) plants. However, non-fagaceous plants were associated with not only arbuscular mycorrhizal fungi but also several clades of ectomycorrhizal (e.g., *Russula*) and root-endophytic ascomycete fungi. Many of the ectomycorrhizal and root-endophytic fungi were detected from both fagaceous and non-fagaceous plants in the community. Interestingly, ectomycorrhizal and arbuscular mycorrhizal fungi were concurrently detected from tiny root fragments of non-fagaceous plants. The plant–fungal associations in the forest were spatially structured, and non-fagaceous plant roots hosted ectomycorrhizal fungi more often in the proximity of ectomycorrhizal plant roots. Overall, this study suggests that belowground plant–fungal symbiosis in subtropical forests is complex in that it includes “non-typical” plant–fungal combinations (e.g., ectomycorrhizal fungi on possibly arbuscular mycorrhizal plants) that do not fall within the conventional classification of mycorrhizal symbioses, and in that associations with multiple functional (or phylogenetic) groups of fungi are ubiquitous among plants. Moreover, ectomycorrhizal fungal symbionts of fagaceous plants may “invade” the roots of neighboring non-fagaceous plants, potentially influencing the interactions between non-fagaceous plants and their arbuscular-mycorrhizal fungal symbionts at a fine spatial scale.

## Introduction

More than 90% of wild terrestrial plant species are estimated to have close ecological interactions with mycorrhizal fungi [1], [2]. These fungal symbionts are essential for the growth and survival of host plants [3]–[5]. For example, mycorrhizal fungi absorb soil nitrogen and/or phosphorus through their extraradical mycelia and transport them to host plants [6], [7]. In return, the hosts provide photosynthetic products (organic carbon) to mycorrhizal fungi [8]. Given that approximately 20% of plant net photosynthetic products are supplied to mycorrhizal fungi [9], [10], mycorrhizal interactions are likely to be major determinants of ecosystem-level properties and processes, such as the productivity of forests, grasslands, and crop lands, and the nutrient and carbon flow between aboveground and belowground biota [11]–[14].

Fungi in plant roots are phylogenetically and ecologically diverse [2]. Arbuscular mycorrhizal fungi in the phylum Glomeromycota are the ancient root symbionts that emerged in the early stages of land plant diversification [15]. They play pivotal roles in nutrient uptake of diverse terrestrial plant species, especially in grasslands and tropical forests [1], [2], [16]. Ectomycorrhizal species in the phyla Ascomycota and Basidiomycota constitute another major group of mycorrhizal fungi [17]. Importantly, members of this group interact with plant clades that often dominate temperate forests (e.g., Pinaceae, Fagaceae, Betulaceae, and Nothofagaceae; [2], [17]). Ectomycorrhizal fungi also interact with tropical trees of Dipterocarpaceae, which are regionally dominant, especially in Southeast Asia [18].

To date, many researchers have investigated the diversity and community composition of mycorrhizal fungi in various types of temperate and tropical forests [19]–[21]. The recent development of high-throughput DNA sequencing (e.g., pyrosequencing; [22], [23]) has enabled the detection of diverse fungal taxa in temperate and tropical forests [18], [24]–[26]. In most studies on mycorrhizal fungal communities, attention has been focused on single functional groups of mycorrhizal fungi because a local predominance of either arbuscular mycorrhizal or ectomycorrhizal plant species often occurs (but see notable exceptions; [27], [28]). Thus, for example, focused investigations have been conducted on ectomycorrhizal fungal community structure on Fagaceae, Betulaceae, or Pinaceae trees in temperate forests (e.g., [24], [29]), arbuscular mycorrhizal fungi on diverse tree species in tropical forests (e.g., [30], [31]), and ectomycorrhizal fungi in Dipterocarpaceae-dominated forests in Southeast Asia [18]. However, in subtropical regions, ectomycorrhizal trees [32] and possibly arbuscular mycorrhizal trees [20] often co-occur in comparable abundances within a single forest [33], and both types of fungal symbionts possibly play important ecological roles (e.g., enhancement of nutrient and carbon cycling) under such circumstances.

Moreover, in addition to mycorrhizal fungi, diverse clades of root-endophytic fungi are known to commonly interact with plants in temperate and Arctic forests [34]–[36], while the diversity and community structure of such endophytes are poorly understood concerning subtropical forests. Although the ecological functions of root-endophytic fungi have yet to be investigated, recent evidence has demonstrated that several clades of these fungi potentially confer benefits to host plants by mineralizing nutrients in the rhizosphere [37] or enhancing the pathogen resistance of host plants [34], [38].

Given the possible involvement of diverse mycorrhizal and endophytic fungi, subtropical forests provide ideal opportunities to investigate how multiple functional groups of root-associated fungi are differentially hosted within a plant community. Furthermore, because those different types of fungi can coexist [36] or compete for space within plant roots [39], [40] (see also [41]), studying such spatial patterns will help us to understand how ecological interactions between fungal symbionts determine the spatial structure of plant–fungal associations in a forest.

To investigate the diversity and spatial structure of belowground plant–fungal symbiosis in a mixed forest of ectomycorrhizal and arbuscular mycorrhizal plants, we conducted a pyrosequencing analysis of root-associated fungi, targeting various functional types of fungi such as arbuscular mycorrhizal, ectomycorrhizal, and endophytic fungi. In the studied forest located in the southern subtropical region of Japan, no single plant species was predominant and both ectomycorrhizal Fagaceae and diverse potentially arbuscular mycorrhizal plants (e.g., Lauraceae, Euphorbiaceae, and Theaceae) co-occurred. Using a high-throughput pyrosequencing analysis of fungal internal transcribed spacer (ITS) sequences, we first determined the community structure of root-associated fungi at the study site. We then compared the community compositions of root-associated fungi between fagaceous and non-fagaceous plant species, and screened for fungal taxa preferentially associated with either of these plant groups. Furthermore, we examined how often different functional groups of fungi were concurrently detected from plant roots and also inferred potential effects of fungus-to-fungus negative interactions from the spatial patterning of belowground plant–fungal associations. Our results provide a basis for understanding how various functional groups of fungi are differentially hosted within a mixed forest of ectomycorrhizal and arbuscular mycorrhizal plants.

## Materials and Methods

### Study Site and Sample Collection

We sampled roots in a subtropical secondary forest located on Yakushima Island, Kagoshima Prefecture, Japan (30.427N, 130.493E, 450 m elevation; granite parent material) in the period November 6–8, 2010. The studied forest is located at the mountainous region on Yakushima Island, where mean annual precipitation exceeds 8,600 mm in some places [42] because of wet seasonal prevailing wind from the sea. In the forest, evergreen tree species of Fagaceae (*Castanopsis sieboldii*, *Lithocarpus edulis*, and *Quercus salicina*) and Lauraceae (e.g., *Machilus japonica* and *Neolitsea sericea*) were common. In addition, various plant species in families such as Apocynaceae, Euphorbiaceae, Rubiaceae, Symplocaceae, Theaceae, and Vitaceae co-occurred. A 29×39-m plot was established and sampling positions were deployed at 1-m intervals (Fig. S1a). We collected root samples from 1,200 sampling positions (30 rows × 40 columns). At each sampling position, we sampled two terminal root fragments (approximately 2 cm long) from the upper part of the A horizon (3 cm below the soil surface). These samples were collected without regard to root morphology or apparent mycorrhizal type. We used this sampling strategy to determine the relative root abundance of plant species in the horizon. Our premise was that the collected root samples as a whole represented the relative frequencies of plant–fungal associations (root–hyphal connections) in the study plot [36], [43], [44]. After collecting root samples, we immediately preserved them in absolute ethanol for storage at –25°C in the laboratory. All necessary permits for the sample collection were issued by Yakushima Forest Ecosystem Conservation Center.

### DNA Extraction, Polymerase Chain Reaction (PCR), and Pyrosequencing

One terminal root sample was randomly selected from each of the 1,200 sampling positions. All soil was carefully removed from each root sample by immersion in 70% ethanol containing 1-mm-diameter zirconium balls followed by shaking for 2 min at a rate of 15×s^–1^ using a TissueLyser II (Qiagen, Venlo, The Netherlands) [36]. Washed roots were frozen at –25°C and then pulverized by shaking at a rate of 20×s^–1^ for 3 min with 4-mm-diameter zirconium balls using a TissueLyser II. Total plant and fungal DNA was extracted from each root sample using the cetyltrimethylammonium bromide (CTAB) method described elsewhere [45].

We analyzed host-plant chloroplast *rbcL* and fungal ITS sequences for each root sample using a tag-encoded massively parallel pyrosequencing procedure [22], [46]. To identify the host plant, we amplified a 0.5-kb *rbcL* gene fragment using the forward primer rbcL_F3 [26] fused with 454-pyrosequencing Adaptor A (5′-CCA TCT CAT CCC TGC GTG TCT CCG ACT CAG-3′) and the 8-mer molecular ID [46]; the reverse primer was rbcL_R4 [26] fused with 454 Adaptor B (5′-CCT ATC CCC TGT GTG CCT TGG CAG TCT CAG-3′). PCR was conducted with a temperature profile of 95°C for 10 min, followed by 40 cycles at 94°C for 20 s, 56°C for 30 s, 72°C for 90 s, and a final extension at 72°C for 7 min using an Ampdirect Plus buffer system (Shimadzu Corp., Kyoto, Japan) and BIOTAQ HS DNA Polymerase (Bioline, London, UK).

To analyze fungal ITS sequences, we amplified the entire ITS region and the partial ribosomal large subunit region using the fungus-specific high-coverage primer ITS1F_KYO2 [47] and the universal primer LR3 (http://www.biology.duke.edu/fungi/mycolab/primers.htm). PCR was conducted with a temperature profile of 95°C for 10 min, followed by 20 cycles at 94°C for 20 s, 50°C for 30 s, 72°C for 120 s, and a final extension at 72°C for 7 min using an Ampdirect Plus buffer system and BIOTAQ HS DNA polymerase. We subjected the PCR product from each root sample to a second PCR step that targeted the ITS2 region. The second PCR was conducted using the universal primer ITS3_KYO2 [47] fused with 454 Adaptor A and sample-specific molecular ID; the reverse universal primer was LR_KYO1b [26] fused with 454 Adaptor B. A buffer system of Taq DNA Polymerase together with Standard Taq Buffer (New England BioLabs, Ipswich, MA, USA) was used with a temperature profile of 95°C for 1 min, followed by 40 cycles at 94°C for 20 s, 50°C for 30 s, 72°C for 60 s, and a final extension at 72°C for 7 min.

The *rbcL* and ITS amplicons obtained were subjected to 454-pyrosequencing. Because of the large sample size, we separately sequenced the first 576 samples, which had been treated in six 96-well PCR plates in the above experimental procedure, and the remaining 624 (96-well plate × 6.5) samples using a GS Junior sequencer (Roche, Basel, Switzerland). We pooled and purified the *rbcL* and ITS amplicons from the first 576 root samples using an ExoSAP-IT cleanup kit (GE Healthcare, Little Chalfont, Buckinghamshire, UK) and a QIAquick PCR Purification Kit (Qiagen). The sequencing of these first 576 samples was conducted according to the manufacturer’s instructions. The amplicons of the remaining 624 samples were pooled and purified, and then sequenced in a second run.

### Assembling of Pyrosequencing Reads

In total, we obtained 94,894 and 103,080 reads for the first and second runs, respectively (DDBJ Sequence Read Archive: DRA001010). We trimmed low-quality 3′ tails with a minimum quality value of 27 from the reads obtained [48]. We discarded *rbcL* reads that were shorter than 400 bp and ITS reads shorter than 150 bp, excluding the forward primer, molecular ID, and ribosomal large subunit positions (for ITS reads only). After the trimming step, 73,405 (16,627 *rbcL* and 56,778 ITS reads) and 83,402 (20,336 *rbcL* and 63,066 ITS reads) reads for the first and second runs, respectively, had passed the quality-filtering process. *RbcL* and ITS reads were recognized by their primer position sequences and analyzed separately. We sorted pyrosequencing reads for each gene based on combinations of sample-specific molecular ID and the pyrosequencing run. The molecular ID and forward primer sequences were removed before the assembling procedure. Denoising of sequence data was performed based on the assembling analysis detailed below (cf. [49]).

For analysis of the host plant *rbcL* gene, we conducted the assembling of the filtered reads using Assams v0.1.2013.01.01 software [36], [50], which is a highly parallelized extension of the accurate and rapid pipeline of the Minimus assembler [51]. Minimus uses standard overlap-layout-consensus algorithm. Overlap is detected by pairwise alignment for each pair of reads. The overlapped reads are multiply-aligned and then majority-rule consensus sequences (i.e., contigs) are generated. Assams first splits the raw input reads into several groups to reduce the number of read pairs in the pairwise alignment process, and the program subsequently assembles reads within each group by Minimus. Assams then make super-contigs by assembling the contigs output from the within-group assembling process. After the super-contig construction, raw input reads are laid out using the super-contigs as guides and then majority-rule consensus sequences are regenerated based on complete-linkage clustering, wherein the radius of the clustering threshold circle is set to one minus a user-given cutoff sequence similarity. Overall, Assams enables accurate assembling as the original Minimus program does, while the former is characterized by its reduced computational loads in the pairwise alignment and parallelized (multi-) threading, allowing much faster processing of pyrosequencing data than the latter.

Using Assams, reads in each sample were assembled with a minimum cutoff similarity of 97%; consensus sequences, which were less likely to contain PCR and pyrosequencing errors than original sequencing reads were, were then obtained for respective samples (hereafter, within-sample consensus sequences). The consensus sequences were subjected to the UCHIME v4.2.40 software [52] (with a minimum score of 0.3 to detect a chimera), to eliminate possible chimeras. Assembling of the within-sample consensus sequences were then performed across samples with a minimum similarity setting of 99.8%. The resulting consensus sequences (hereafter, among-sample consensus sequences) were BLAST-searched [53] to refer to *rbcL* sequences in the NCBI nucleotide database (http://www.ncbi.nlm.nih.gov/) and host plant species were identified based on the BLAST results, taking into account the plant species composition observed in the studied forest.

In our analysis of the fungal ITS2 region, we subjected 119,844 reads (56,778 from the first run and 63,066 from the second run) to detection and removal of chimeras using UCHIME software after obtaining within-sample consensus sequences based on assembling with a minimum cutoff similarity of 97%. Of the 119,844 ITS reads, 790 were discarded as chimeras, leaving a total of 119,054 reads. Because fungal ITS2 sequences sometimes have more than 3% intraspecific variation [54], the minimum cutoff similarity for the among-sample assembling process was set to 95% in Assams software (Data S1). Using 97% similarity cutoff in the among-sample assembling (Data S2–5) did not qualitatively change the downstream ecological analyses (Fig. S2).

The resulting among-sample consensus sequences represented fungal operational taxonomic units (OTUs; Data S1). Of the 119,054 reads, 566 were removed as singletons. On average, 98.7 (SD = 62.2; *N* = 1200) ITS reads, excluding singletons, were obtained for each sample, and the mean number of OTUs per sample was 8.8 (SD = 6.3). The rarefaction curves, which were drawn using the vegan v.2.0-2 package [55] of R (http://cran.r-project.org/), showed that the number of OTUs was not saturated for many of the samples (Fig. S1b), although many of rare OTUs might represent contaminants rather than actual fungal symbionts of the samples.

Because sequences of rare OTUs are likely to contain a high proportion of pyrosequencing errors, we excluded OTUs consisting of less than five reads in every sample from the following analyses. Samples with fewer than 20 high-quality reads were excluded in the following analyses. Both *rbcL* and ITS data were available for 849 samples.

### Taxonomic Assignment of Fungal OTUs

To infer the taxonomy of respective ITS OTUs, we prepared a local BLAST database based on the “nt” database downloaded from the NCBI ftp server (http://www.ncbi.nlm.nih.gov/Ftp/) on November 18, 2012. The “nt” database was filtered by removing sequences without taxonomic information at the genus level. Based on the filtered “nt” database, taxonomic assignment of the OTUs was performed based on the Query-centric auto-*k*-nearest neighbor (QCauto) method [56], which were known to return the most accurate taxonomic identification results among the existing methods of automated DNA barcoding [56], using the program Claident v0.1.2012.11.23 [56], [57]. The benefit of using the QCauto method is that it enables the accurate and fully-automated taxonomic identification based on BLAST+ searches [58] without setting any arbitrary threshold of sequence identity percentages or E-values [56]. In the taxonomic identification process, the relaxed lowest common ancestor algorithm [36], [59] was used. Based on the taxonomic assignment, we classified OTUs into ectomycorrhizal fungi, arbuscular mycorrhizal fungi, and fungi with unknown ecological functions. In our screening of ectomycorrhizal fungi, we referred to the review of the taxa belonging to the functional group [17]: fungal genera or families that were predominantly ectomycorrhizal were putatively designated as ectomycorrhizal.

### Data Matrices and Fungal Diversity

For each of the 849 samples with plant and fungal information, we determined the presence/absence of fungal OTUs. In this process, only OTUs with more than 5% of the sample total reads were designated as present in a sample (Data S3) to remove rare OTUs, which can be contaminants. Rarefaction methods are commonly used to convert next-generation sequencing datasets into community ecological data matrices, but the problems regarding possible contaminants in next-generation sequencing may not be solved by rarefaction approaches: note that the abovementioned 5%-cutoff treatment and rarefaction-based treatment (resample size = 50 reads/sample; sample size after rarefaction = 732; Data S4; Fig. S3) yielded qualitatively similar results in the following community ecological analyses (Figs. S4 and S5). The number of ITS reads retained after the 5%-cutoff treatment was 78,949, and the mean number of sequences and OTUs per sample was 93.0 (SD = 45.5) and 3.58 (SD = 1.75), respectively. After the 5%-cutoff treatment, a binary matrix depicting the presence or absence of OTUs in each sample was constructed (Data S4; hereafter, “sample-level” matrix). The taxonomic diversity of fungi in the matrix was evaluated by the number of OTUs that belonged to each taxon at the phylum, order, or genus level.

The “sample-level” matrix was used to construct another matrix that expressed associations between plant species and fungal OTUs (Data S5: hereafter, “plant × fungal” matrix). In the plant × fungal matrix, row *i* represented a plant species *i*, column *j* represented a fungal OTU *j*, and the value in each matrix cell (*A_ij_*) represented the number of root samples in which the focal plant–fungal association was observed. Subsequently, the proportion of a plant–fungal association (combination) in the matrix (*P_ij_*) was estimated as *P_ij_* = *A_ij_*/∑*_i_*∑*_j_ A_ij_*.

The proportions of plant–fungal associations was also calculated separately for fagaceous plant species, which were considered to be primarily ectomycorrhizal according to the conventional classification of mycorrhizal symbiosis [1], [2] (but see [60]), and for the remaining (non-fagaceous) plant species, many of which were potentially arbuscular mycorrhizal but detailed mycorrhizal properties of them remained to be clarified [1], [61]. As a result, we obtained the proportion of associated fungi for fagaceous plants (*P_fagaceous, j_* = *A_fagaceous, j_*/∑*_fagaceous_*∑*_j_ A_ij_*) and that for non-fagaceous plants (*P_non-fagaceous, j_* = *A_non-fagaceous, j_*/∑*_non-fagaceous_*∑*_j_ A_ij_*). These proportions of associated fungi were visualized separately for fagaceous and non-fagaceous plants at the phylum, order, and genus levels (see Data S6 for the used matrices of *A_fagaceous, j_* and *A_non-fagaceous, j_*).

Concomitantly, to compare the proportions of associated fungi between fagaceous and non-fagaceous plants, a *G*-test [62] was conducted at each of the phylum, order, and genus levels. Note that the approximation to theoretical chi-squared distribution is better for *G*-test than for Pearson’s chi-squared test. The proportions of associated fungi were also examined by classifying fungal OTUs into ectomycorrhizal, arbuscular mycorrhizal, and the remaining fungi. Since the ecological impacts of non-mycorrhizal fungal symbionts on the host plant growth/survival are poorly known, we hereafter refer to this third group of fungi as those with unknown ecological functions.

### Fungi on Fagaceous and Non-fagaceous Plants

To statistically screen for fungal OTUs that preferentially associated with fagaceous plant species and OTUs that preferentially associated with non-fagaceous plant species, we performed a multinomial species classification using the CLAM test [63]. In the CLAM test, fungal OTUs’ preferential associations with plants were evaluated using a multinomial model based on the estimated relative abundance of fungal OTUs on two types of host plants. Using this model, we classified fungal OTUs into the following categories: fungi preferentially associated with fagaceous plants, fungi preferentially associated with non-fagaceous plants, fungi commonly associated with both fagaceous and non-fagaceous plants, and fungi that were rare on both types of host plants. The CLAM test was performed based on Data S4, in which the sample-level matrix and the host plant information of respective samples are provided, using the vegan package with a specialization threshold value of 2/3 [63].

We further examined the way in which ectomycorrhizal and arbuscular mycorrhizal fungi were differentially shared among plant species. The numbers of shared ectomycorrhizal fungal OTUs and arbuscular mycorrhizal OTUs were calculated for each pair of plant species based on the plant × fungal matrix (Data S5).

### Co-existence of Multiple Fungal Functional Groups in Roots

We examined the prevalence of colonization by multiple fungal functional groups in the terminal roots of plants in the subtropical forest investigated. For each root sample, we determined the presence of ectomycorrhizal and arbuscular mycorrhizal fungi and the presence of fungi with unknown ecological functions. Subsequently, we separately calculated the proportion of the root samples colonized by multiple functional groups of fungi (e.g., colonization by both ectomycorrhizal and arbuscular mycorrhizal fungi) for fagaceous and non-fagaceous plants.

### Effects of Spatial Proximity to Fagaceous Plants on the Fungal Community Composition of Non-fagaceous Plants

Given that diverse ectomycorrhizal fungi were detected from the roots of non-fagaceous plant species (see Results), which had not been considered as ectomycorrhizal [1], we evaluated the effects of spatial proximity to fagaceous plants on the fungal community composition of non-fagaceous plant species. We first evaluated whether or not the community composition of root-associated fungi were spatially auto-correlated within the 29 × 39-m study plot based on a Mantel correlogram analysis. Mantel’s correlation between the dissimilarity of root-associated fungal OTU composition and the Euclidean distance spanning sampling positions was evaluated at each distance class using R (1,000 randomizations). In the analysis, the dissimilarity of fungal OTU composition between root samples was evaluated by Raup-Crick *β*-diversity [64].

We then tested whether or not the fungal community composition of non-fagaceous plant roots were influenced by the adjacency of fagaceous plants. In the analysis, we first classified the root samples of non-fagaceous plants into two categories in terms of the presence or absence of fagaceous plants at adjacent sampling positions. Non-fagaceous plant samples with less than three adjacent sampling positions with sequence data (Fig. S1a) were excluded from the subsequent analysis. Based on Fisher’s exact test, we tested whether or not the proportions of the non-fagaceous root samples colonized by ectomycorrhizal fungi differed depending on the presence/absence of fagaceous plants at adjacent sampling locations. An additional Fisher’s exact test was conducted to test whether or not spatial proximity to fagaceous plants affected the proportion of the non-fagaceous root samples colonized by arbuscular mycorrhizal fungi.

## Results

### Plant and Fungal Diversity in Root Samples

The pyrosequencing analysis of the chloroplast *rbcL* gene detected 36 plant species in 849 terminal root samples (Fig. S1c). Among them, the fagaceous tree *C. sieboldii* was the most common; other deciduous broad-leaved trees (e.g., *Vernicia cordata* and *Styrax japonica*) and a woody vine (*Trachelospermum asiaticum*) were also common. At the family level, Fagaceae (*Castanopsis*, *Quercus*, and *Lithocarpus*) and Lauraceae (*Neolitsea*, *Litsea*, and *Machilus*) made up 25.2% and 18.8%, respectively, of the 849 root samples. Among the plants, only the three fagaceous species, *C. sieboldii*, *Q. salicina*, and *L. edulis*, were presumably ectomycorrhizal.

From the 849 sequenced terminal-root samples, we obtained 580 ITS OTUs, excluding possible chimeras, non-fungal sequences, the OTUs representing less than five pyrosequencing reads in every sample, and the OTUs representing less than 5% of sample-total reads (Data S3). Of the 580 OTUs detected, 279 (48.1%) were Ascomycota, 219 (37.8%) were Basidiomycota, 58 (10.0%) were Glomeromycota, three (0.5%) were Chytridiomycota, and 21 (3.6%) were unidentifiable at the phylum level (Fig. S6a). At the order level, the Russulales (32.9%), Agaricales (9.8%), Glomerales (8.1%), and Helotiales (5.0%) were the most common (Fig. S6b). At the genus level, ectomycorrhizal genera such as *Russula* (9.3%), *Tomentella* (2.1%), *Sebacina* (1.9%), *Lactarius* (1.7%), and *Elaphomyces* (0.7%) were detected, and arbuscular mycorrhizal genera such as *Glomus* (2.6%) and *Rhizophagus* (1.0%) were also present (Fig. S6c). Of the 580 fungal OTUs, 132 (22.8%) were presumed to be ectomycorrhizal and 58 (10.0%) to be arbuscular mycorrhizal; the ecological functions of the remaining 390 OTUs (67.2%) were not assigned (Fig. S6d; Data S3).

### Comparison of the Proportions of Associated Fungi between Fagaceous and Non-fagaceous Plants

**Figure 1:**
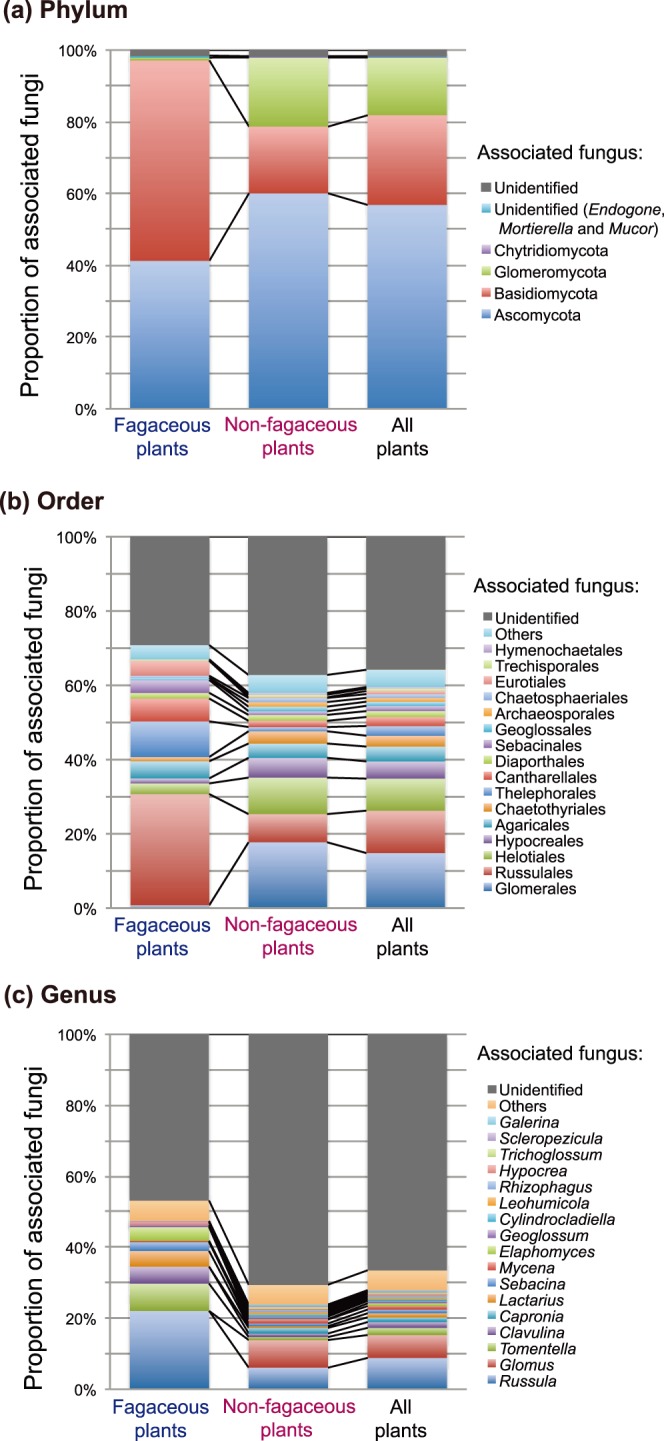
Comparison of the proportions of associated fungi between fagaceous and non-fagaceous plant species. (a) Proportion of plant–fungal association (*P_ij_*) at the phylum level. The proportions of associated fungal taxa are shown for fagaceous (214 root samples), non-fagaceous (635 root samples), and all (fagaceous+non-fagaceous; 214+635 = 849 root samples) plant species. In total, 521, 2516, and 3037 plant–fungal associations were observed for fagaceous, non-fagaceous, and all plant species, respectively. (b) Proportion of plant–fungal association (*P_ij_*) at the order level. (c) Proportion of plant–fungal association (*P_ij_*) at the genus level.

The proportions of associated fungi differed significantly between fagaceous and non-fagaceous plants at each of the phylum (*G* = 353.0, df = 5, *P*<0.0001), order (*G* = 496.7, df = 39, *P*<0.0001), and genus (*G* = 394.3, df = 74, *P*<0.0001) levels (Fig. 1). At the phylum level, associations with Basidiomycota were most common for fagaceous plants (55.9%), while associations with Ascomycota were most common for non-fagaceous plants (60.1%; Fig. 1a). Furthermore, only 0.8% of fagaceous plant–fungal associations involved Glomeromycota, while the arbuscular mycorrhizal fungal taxa accounted for 19.2% of the plant–fungal associations among non-fagaceous plants (Fig. 1a). At the order level, associations with fungi of Russulales and Thelephorales were common among fagaceous plants (29.9% and 9.6%, respectively), but not among non-fagaceous plants (7.6% and 1.2%, respectively; Fig. 1b). In contrast, associations with fungi of Glomerales, Helotiales, and Hypocreales were common among non-fagaceous plants (17.7%, 9.8%, and 5.3%, respectively), but associations with fungi of these orders were rare among fagaceous plants (Fig. 1b). At the genus level, ectomycorrhizal taxa such as *Russula*, *Tomentella*, *Clavulina*, and *Lactarius* accounted for 22.1%, 7.7%, 4.8%, and 4.4%, respectively, of the plant–fungal associations for fagaceous plants (Fig. 1c). For non-fagaceous plants, associations with the arbuscular mycorrhizal genus *Glomus* were most common (7.8%); we did not detect associations with this genus for fagaceous plants (Fig. 1c).

**Figure 2:**
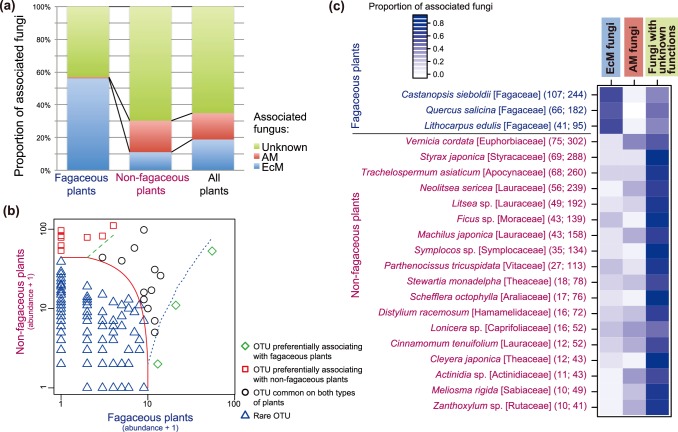
Relationship between plant and fungal functional groups. (a) Proportion of plant–fungal association (*P_ij_*) in terms of fungal functional groups. Results are shown for fagaceous, non-fagaceous, and all (fagaceous+non-fagaceous) plant species. In total, 521, 2516, and 3037 plant–fungal associations were observed for fagaceous, non-fagaceous, and all plant species, respectively. EcM, ectomycorrhizal fungi; AM, arbuscular mycorrhizal fungi; Unknown, fungi with unknown ecological functions. (b) CLAM analysis of fungi on fagaceous and non-fagaceous plants. Fungal OTUs were classified into the following categories: fungi preferentially associated with fagaceous plants (diamond), fungi preferentially associated with non-fagaceous plants (square), fungi commonly associated with both fagaceous and non-fagaceous plants (circle), and fungi that were rare in both types of host plants (triangle). (c) Proportions of associated fungal functional groups for each plant species. The proportions of associations that involved EcM and AM fungi as well as fungi with unknown ecological functions are shown for each plant species with 10 or more root samples. The number of root samples (left) and the number of the plant–fungal associations observed in the root samples (right) are shown in parentheses: the latter was used in the calculation of the proportions of associated fungi.

A further analysis indicated that the proportions of associated fungal functional groups differed significantly between fagaceous and non-fagaceous plants (*G* = 546.6, df = 2, *P*<0.0001; Fig. 2a). Among ectomycorrhizal plants, 56.2% of the plant–fungal associations involved ectomycorrhizal fungi, while the proportion of plant–ectomycorrhizal fungal associations for non-fagaceous plants was 11.1% (Fig. 2a). In contrast, associations with arbuscular mycorrhizal fungi were common among non-fagaceous plants (19.2%), but only 0.8% of plant–fungal associations involved arbuscular mycorrhizal fungi for fagaceous plants (Fig. 2a).

### Fungi on Fagaceous and Non-fagaceous Plants

Among 580 fungal OTUs examined in the CLAM test, only three preferentially associated with fagaceous plants (Fig. 2b). Of these, one was in the ectomycorrhizal fungal genus *Tomentella* (OTU 801; Table 1), and the others were unidentified ascomycete fungi (Data S3). Eight fungal OTUs were preferentially associated with non-fagaceous plants (Fig. 2b; Table 1); these included arbuscular mycorrhizal fungi (OTUs 49, 1871, and 1211) and members of Helotiales (OTU 1721) and Nectriaceae (OTU 1689) (Table 1). In contrast to fungi with preferences for plants, 12 of the fungal OTUs were commonly associated with both fagaceous and non-fagaceous plants (Fig. 2b; Table 1; Data S3). Of these 12 taxa, seven were ectomycorrhizal taxa (*Russula*, *Clavulina*, and *Tomentella*; Table 1; Data S3); the remaining OTUs included a Helotiales OTU, two OTUs in the class Sordariomycetes, and two unidentified ascomycete OTUs (Data S3).

**Table 1:** Fungal OTUs commonly detected from plant roots in the subtropical forest.

	Identification results based on the QCauto method				*N* (associations)				CLAM test	BLAST top-hit				
OTU	Phylum	Order	Family	Genus	Fagaceous plant	Non-fagaceous plant	Total	Functional group		Description (order)	Coverage	E value	Identity	Accession
49	Glomeromycota	Glomerales	Glomeraceae		3	110	113	AM	Non-fagaceous plants	Glomeromycetessp. (n.a.)	99%	8.0E-136	94%	JQ272369.1
773	Ascomycota				54	52	106	Unknown	Fagaceous plants	Hyaloscyphaceae sp. (Helotiales)	99%	2.0E-136	97%	JQ272392.1
1719	Ascomycota				8	97	105	Unknown	Both plants	*Sphaerosporella* sp. (Pezizales)	99%	9.0E-51	81%	JQ711781.1
1721	Ascomycota	Helotiales			0	95	95	Unknown	Non- fagaceous plants	Helotiales sp. (Helotiales)	99%	5.0E-148	99%	GU166468.1
1691	Ascomycota				2	81	83	Unknown	Non- fagaceous plants	Sordariomycetessp. (n.a.)	99%	3.0E-150	99%	JX243935.1
137	Ascomycota				0	82	82	Unknown	Non- fagaceous plants	Ascomycota sp. (n.a.)	86%	5.0E-138	97%	HQ623460.1
1689	Ascomycota	Hypocreales	Nectriaceae		1	78	79	Unknown	Non- fagaceous plants	*Neonectria* sp. (Hypocreales)	99%	4.0E-159	100%	JX243941.1
1871	Glomeromycota	Glomerales	Glomeraceae	*Glomus*	0	78	78	AM	Non- fagaceous plants	*Glomus* sp. (Glomerales)	99%	8.0E-156	97%	AJ504624.1
1609	Ascomycota	Helotiales			6	57	63	Unknown	Both plants	*Meliniomyces vraolstadiae* (Helotiales)	99%	1.0E-129	96%	HQ157884.1
1211	Glomeromycota	Glomerales	Glomeraceae	*Glomus*	0	61	61	AM	Non- fagaceous plants	*Glomus* sp. (Glomerales)	100%	8.0E-141	94%	HE794038.1
1755	Ascomycota				0	53	53	Unknown	Non- fagaceous plants	Ascomycota sp. (n.a.)	80%	2.0E-122	95%	JN596345.1
1415	Ascomycota				2	43	45	Unknown	Both plants	*Sphaerosporella* sp. (Pezizales)	100%	2.0E-62	84%	JQ711781.1
1653	Ascomycota	Diaporthales	Valsaceae		4	39	43	Unknown	Both plants	*Phomopsis* sp. (Diaporthales)	99%	2.0E-156	99%	HM751804.1
1727	Ascomycota				11	30	41	Unknown	Both plants	*Lecythophora hoffmannii* (Coniochaetales)	99%	7.0E-67	83%	AB231012.1
1159	Basidiomycota	Russulales	Russulaceae	*Russula*	13	25	38	EcM	Both plants	*Russula* aff. *alboareolata* (Russulales)	100%	1.0E-169	95%	AB509481.1
925	Ascomycota	Chaetothyriales	Herpotrichiellaceae	*Capronia*	0	38	38	Unknown	-	*Capronia* sp. (Chaetothyriales)	100%	2.0E-146	97%	AF284128.1
801	Basidiomycota	Thelephorales	Thelephoraceae	*Tomentella*	20	10	30	EcM	Fagaceous plants	*Thelephoraceae* sp. (Theleophorales)	100%	8.0E-166	96%	JF273547.1
1605	Ascomycota				0	28	28	Unknown	–	Helotiales sp. (Helotiales)	99%	5.0E-123	95%	JX243904.1
2525	Basidiomycota	Russulales	Russulaceae	*Russula*	10	16	26	EcM	Both plants	*Russula sororia* (Russulales)	100%	0.0E+00	100%	AB531460.1
811	Ascomycota	Helotiales			0	26	26	Unknown	–	*Meliniomyces* sp. (Helotiales)	100%	5.0E-113	93%	EF093175.1
1063	Ascomycota				0	23	23	Unknown	–	*Caloplaca monacensis* (Teloschistales)	100%	3.0E-75	84%	HM538502.1
1113	Basidiomycota	Russulales	Russulaceae	*Russula*	8	15	23	EcM	Both plants	*Russula* sp. (Russulales)	100%	0.0E+00	99%	AB509982.1
805	Glomeromycota	Glomerales	Glomeraceae		0	23	23	AM	–	*Glomus* sp. (Glomerales)	100%	1.0E-74	82%	AJ504640.1
1773	Ascomycota				0	21	21	Unknown	–	Helotiales sp. (Helotiales)	99%	6.0E-112	92%	JX852365.1
1845	Basidiomycota	Russulales	Russulaceae		8	12	20	EcM	Both plants	*Arcangeliella camphorata* ()	100%	0.0E+00	96%	EU846241.1

The ID numbers of fungal OTUs and the number of terminal root samples in which respective fungi were observed are shown. The results of molecular identification based on the QCauto method [56] and manual-BLAST searches are shown for each OTU. Fungal OTUs that appeared in 20 or more root samples are shown in decreasing order. Ectomyocrrhizal (EcM) and arbuscular mycorrhizal (AM) fungi are indicated at the column “Functional group”. The result of the CLAM test is also indicated (see Fig. 2b).

In Figure 2c, the proportions of associations involving ectomycorrhizal fungi, arbuscular mycorrhizal fungi, and fungi with unknown ecological functions are shown for each plant species represented in 10 or more root samples. Among fagaceous plant species, 54.4–57.4% of plant–fungal associations involved ectomycorrhizal fungi, but only 0.0–1.2% involved arbuscular mycorrhizal fungi (Fig. 2c). For non-fagaceous plant species, the proportion of associations involving ectomycorrhizal fungi ranged from 4.9% to 20.4% (Fig. 2c). For these non-fagaceous plant species, associations with arbuscular mycorrhizal fungi (2.2–38.7%) and fungi with unknown ecological functions (48.1–86.8%) occurred more often than for fagaceous plants (0.0–1.2% and 41.4–45.6%, respectively; Fig. 2c).

**Figure 3:**
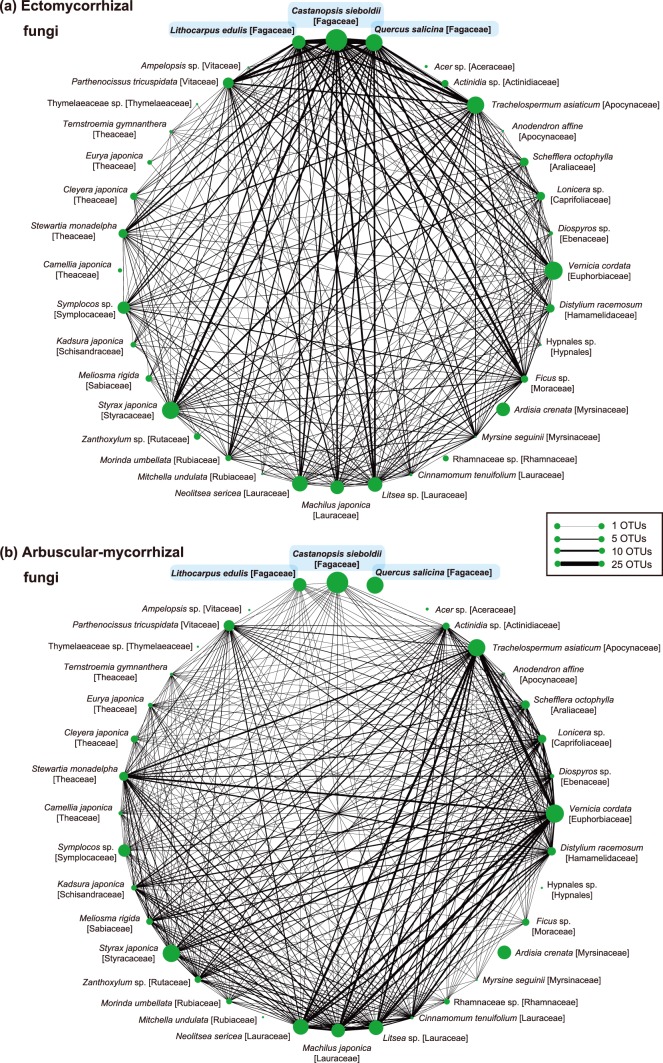
Number of ectomycorrhizal and arbuscular mycorrhizal fungal OTUs shared among plant species. (a) Number of shared ectomycorrhizal fungal OTUs. The line thickness is proportional to the number of fungal OTUs shared in each pair of plant species. The size of circles roughly represents the relative abundance of plant species that was evaluated by the number of root samples (Fig. S1c). (b) Number of shared arbuscular mycorrhizal OTUs.

Our analysis of the number of fungal OTUs shared between plant species further demonstrated that ectomycorrhizal fungi commonly associated with not only fagaceous plants but also with various non-fagaceous plants (Fig. 3a). In total, 27 or 21 ectomycorrhizal fungal OTUs were shared between *C. sieboldii* and each of the other fagaceous species. The *Castanopsis* species also shared 17 and 14 ectomycorrhizal fungal OTUs with *T. asiaticum* and *Parthenocissus tricuspidata*, respectively (Fig. 3a). In contrast, arbuscular mycorrhizal fungi were shared mainly among non-fagaceous plant species (Fig. 3b). For example, 16 and 14 arbuscular mycorrhizal OTUs were shared between the woody vine *T. asiaticum* and the deciduous tree *V. cordata*, and between the broad-leaved trees *M. japonica* and *V. cordata*, respectively (Fig. 3b). See Figure S7 for the number of all functional groups of fungal OTUs that were shared among plant species.

### Co-existence of Multiple Fungal Functional Groups in Roots

**Figure 4:**
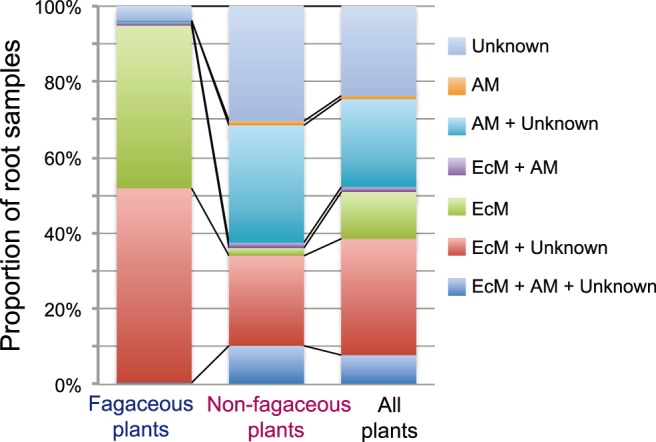
Co-existence of multiple fungal functional groups in roots. For each root sample, the presence of ectomycorrhizal (EcM) and arbuscular mycorrhizal (AM) fungi as well as that of fungi with unknown ecological functions were evaluated. The proportion of root samples colonized by single or multiple fungal functional group(s) is shown for fagaceous (214 roots), non-fagaceous (635 roots), and all (fagaceous+non-fagaceous; 849 roots) plant species.

Among fagaceous plants, 94.4% of the root samples hosted ectomycorrhizal fungi but not arbuscular mycorrhizal fungi (Fig. 4). Co-existence of ectomycorrhizal and arbuscular mycorrhizal fungi was observed in only 1.4% of the fagaceous plant root samples. Among non-fagaceous plants, 32.1% of the root samples hosted arbuscular mycorrhizal fungi but no ectomycorrhizal fungi (Fig. 4). Co-existence of arbuscular mycorrhizal and ectomycorrhizal fungi was observed in 11.7% of the non-fagaceous plant samples. Given that the number of sequencing reads obtained per sample was relatively small (98.7 reads on average) due to the limitation of highly-parallelized pyprosequencing design (Fig. S1b), the actual rate of the co-occurrence of arbuscular mycorrhizal and ectomycorrhizal fungi in roots may be even higher than that observed in the present analysis. The analysis also showed that root samples of non-fagaceous plants were ubiquitously colonized by fungal OTUs with unknown ecological functions (Fig. 4).

### Effects of Spatial Proximity to Fagaceous Plants on the Fungal Community Composition of Non-fagaceous Plants

**Figure 5:**
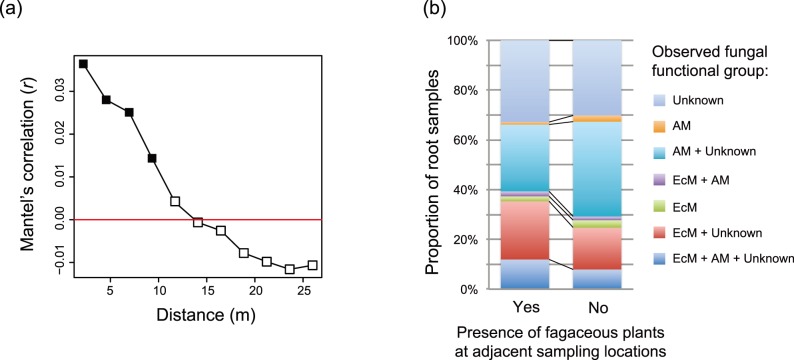
Spatial structure of plant–fungal associations. (a) Spatial autocorrelation of root-associated fungal OTU composition. At each distance class, Mantel’s correlation between spatial distance and dissimilarity of root-associated fungal composition (Raup-Crick *β*-diversity) was examined. Filled squares represent statistically significant correlation (*P*<0.05) after Bonferroni correction. See Figure S8 for the results of the additional spatial autocorrelation analyses applied separately for fagaceous-plant and non-fagaceous-plant root samples. (b) Spatial structure and the co-existence of multiple fungal functional groups in roots. For each root sample, the presence of ectomycorrhizal (EcM) and arbuscular mycorrhizal (AM) fungi as well as that of fungi with unknown ecological functions were evaluated. The proportion of non-fagaceous root samples colonized by single or multiple fungal functional group(s) was calculated under two alternative situations: fagaceous plants were present at adjacent sampling locations in one (left; 201 root samples), but were absent in the other (right; 202 root samples).

At the study site, the composition of fungi in root samples was spatially auto-correlated within the scale of <10 m (Figs. 5a and S8). As expected from the spatial autocorrelation, non-fagaceous plant roots were colonized by ectomycorrhizal fungi more frequently in the presence of adjacent fagaceous plants (Fisher’s exact test; odds ratio = 1.57, *P* = 0.036; Fig. 5b). On the other hand, the effects of proximity to fagaceous plants were non-significant regarding associations between non-fagaceous plants and arbuscular mycorrhizal plants (odds ratio = 0.719, *P = *0.11; Fig. 5b).

## Discussion

In the subtropical forest studied, the composition of fungal root symbionts was significantly different between fagaceous and non-fagaceous plant species (Fig. 1). Among fagaceous plants, ectomycorrhizal fungi were major symbionts in the roots, while non-fagaceous plants frequently associated with arbuscular mycorrhizal fungi (Figs. 1 and 2; Table 1). Thus, fagaceous and non-fagaceous plants hosted different compositions of mycorrhizal fungi in the target subtropical forest, as expected from the conventional classification of mycorrhizal symbioses [2].

However, such compartmentalization of mycorrhizal symbioses was incomplete in the forest investigated (Fig. 3). Specifically, ectomycorrhizal and arbuscular mycorrhizal fungi sometimes co-occurred on the same plant species (Fig. 2), even within the same 2-cm fragment of terminal root (Fig. 4). Several plant species are already known to host both ectomycorrhizal and arbuscular mycorrhizal fungi [27], [60], [65], [66]. For example, the ectomycorrhizal basidiomycete fungus *Tricholoma matsutake* is able to form unique intra- and intercellular structures in the roots of a primarily arbuscular mycorrhizal plant, *Cedrela odorata* (Meliaceae) [66]. Likewise, roots of the oak species *Quercus rubra* are colonized by both ectomycorrhizal and arbuscular mycorrhizal fungi in natural environments [60]. Intriguingly, *Q. rubra* seedlings planted near an arbuscular mycorrhizal plant species are often colonized by arbuscular mycorrhizal fungi, while *Q. rubra* seedlings located near another oak species usually host ectomycorrhizal fungi [60]. These observations suggest that mycorrhizal associations can be plastic in nature; “non-typical” combinations of plant and fungal mycorrhizal types may occur in mixed forests of ectomycorrhizal and arbuscular mycorrhizal plant species. The non-typical plant–fungal associations observed in the present study (Figs. 2–4) may reflect such a possible complexity in mycorrhizal interactions, although another possibility that fungal hyphae are merely adhering to the roots of non-typical hosts should be explored as well.

Our data also suggested that while associations with both ectomycorrhizal and arbuscular mycorrhizal fungi may occur for non-fagaceous plants, this was rarely the case for fagaceous plants in the forest studied (Fig. 2). Several ectomycorrhizal fungal OTUs, especially those in the genera *Russula* and *Clavulina*, occurred commonly on both fagaceous and non-fagaceous plant species (Fig. 2b; Table 1; Data S3). In contrast, arbuscular mycorrhizal fungi were observed almost exclusively on non-fagaceous plants in our samples (Fig. 2b; Table 1; Data S3). Although difference in belowground mycelial densities of ectomycorrhizal and arbuscular mycorrhizal fungi can generate the pattern, the observed asymmetric distribution of the two types of mycorrhizal fungi is intriguing, given that a previous study in a temperate forest reported the opposite pattern, i.e., colonization by arbuscular mycorrhizal fungi on an oak species (see above; [60]). Thus, such co-occurrence patterns of arbuscular-mycorrhizal and ectomycorrhizal fungi in roots may depend on local biotic/abiotic environmental conditions.

Given that the co-occurrence of ectomycorrhizal and arbuscular mycorrhizal fungi was observed even within a tiny root fragment of non-fagaceous plants (Fig. 4), some kinds of ecological interactions between the two types of mycorrhizal fungi may partly responsible for the observed patterns of plant–fungal associations. For example, ectomycorrhizal fungal symbionts of fagaceous plants may “invade” the roots of neighboring non-fagaceous plants and thereby sometimes co-occur with arbuscular mycorrhizal fungi at the fine spatial scale.

However, interactions between ectomycorrhizal and arbuscular mycorrhizal fungi in roots may be negative (or competitive) and, as a consequence, ectomycorrhizal fungi may expel arbuscular mycorrhizal fungi from non-fagaceous plant hosts under certain root environmental conditions [67] (see also [41], [68]). Thus, we hypothesize that by competitively excluding arbuscular mycorrhizal fungi from adjacent non-fagaceous plant individuals, ectomycorrhizal fungi and their fagaceous plant hosts may obtain more soil nutrients, thereby establishing advantage over their neighboring competitors.

To conduct a preliminary analysis on this hypothesis, we first tested whether or not spatial proximity to fagaceous plants affected the rate of associations between non-fagaceous plants and ectomycorrhizal fungi. Such colonization of ectomycorrhizal fungi to neighboring non-fagaceous plants was expected to occur, because plant–fungal associations were spatially auto-correlated within the study plot (Fig. 5a and S8). As expected, non-fagaceous plants hosted ectomycorrhizal fungi more frequently in the presence of fagaceous plants at adjacent sampling locations (Fig. 5b). This result leads to the further prediction that ectomycorrhizal fungi competitively exclude arbuscular mycorrhizal fungi from adjacent non-fagaceous plant roots. This prediction was not supported in the present dataset (Fig. 5b). However, given its potential impacts on the entire structure of plant–fungal symbiosis in a forest, the roles of fungus-to-fungus negative interactions [39]–[41] in the spatial structuring of belowground plant–fungal interactions deserve further field-observational and experimental investigations.

The present study also showed that diverse clades of non-mycorrhizal (or endophytic) fungi were involved in the belowground plant–fungal associations of the subtropical forest investigated (Figs. 2–4; Table 1). The involvement of non-mycorrhizal fungi was conspicuous for non-fagaceous plants (Table 1; see also Figs. 2–4). In most studies on primarily arbuscular mycorrhizal plants, Glomeromycota-specific primers have been used to examine the diversity of fungal symbionts in roots [25], [43]. The use of universal primers [47] in the present study allowed us to detect diverse root-associated fungi in addition to the 58 OTUs of arbuscular mycorrhizal (Glomeromycota) fungi (Fig. S6). Among fungi with poorly known ecological functions, we commonly found ascomycetes in the orders Helotiales, Hypocreales, and Chaetothyriales (Table 1), but many other ascomycete OTUs remained unidentified, even at the order level (see the low BLAST identity scores for OTUs 1719, 1415, 1727, and 1063 in Table 1). Root-associated fungi in these ascomycete orders have been commonly observed in temperate and Arctic regions [34], [36], [37], and several clades of such non-mycorrhizal fungi presumably promote the growth and/or survival of plant hosts by, e.g., mineralizing organic nitrogen in rhizosphere [34], [37], [38]. Given that about 30% of non-fagaceous plant root samples included fungi with unknown functions but no mycorrhizal fungi (Fig. 4), some kinds of endophytic fungi (e.g., dark septate endophytes; [37]) might be working as mutualistic symbionts by enhancing host plants’ nutritional conditions in the subtropical forest.

As mentioned above, our high-throughput pyrosequencing analysis revealed a number of non-typical plant–fungal associations as well as the underappreciated co-occurrence of ectomycorrhizal and arbuscular mycorrhizal fungi within tiny root fragments. Nevertheless, these poorly recognized patterns should be interpreted with caution because pyrosequencing-based DNA barcoding provide no information about the physiological status of each root–hyphal contact [69]. In other words, the present data may include plant–fungal associations in which fungal hyphae are merely attached to roots with no physiological effects on their hosts. Thus, detailed microscopic observations of dissected roots [70], [71] and transcriptomic analyses of plant/fungal genes expressed in mycorrhizae [72], [73] are required for better understanding the contribution of such non-typical plant–fungal associations to plant growth or survival.

## Supporting Information

Figure S1 Sampling locations and the summary of the pyrosequencing. (a) Distribution of fagaceous and non-fagaceous root samples at the study plot. Root samples were collected at 1-m intervals. Filled and open circles represent fagaceous and non-fagaceous root samples, respectively. See Data S3 for detailed information of each root sample. (b) Rarefaction curve of the number of OTUs in each root sample against the number of pyrosequencing reads excluding singletons. (c) Composition of host plant species identified by chloroplast *rbcL* sequences. The proportion of each plant species among the 849 root samples is shown.
(PDF)

Figure S2 Results of community ecological analyses based on 97% cutoff similarity for the assembling of fungal OTUs. Additional statistical analyses were conducted based on the 97% cutoff similarity setting of ITS sequences. The exclusion of the OTUs that represented 5% or less of sample total reads (see text) was not applied in this additional analysis to confirm the robustness of the downstream statistical results to alternative data treatments. Compare the results on 97% cutoff similarity (this supplementary figure) with those on 95% cutoff similarity (Figs. 1 and 2). (a) Proportion of plant–fungal association (*P_ij_*) at the phylum level. Results are shown for fagaceous (214 root samples), non-fagaceous (635 root samples), and all (fagaceous+non-fagaceous; 214+635 = 849 root samples) plant species. In total, 895, 6562, and 7457 plant–fungal associations were observed for fagaceous, non-fagaceous, and all plant species, respectively. The proportions of associated fungi were significantly different between fagaceous and non-fagaceous plants (*G*-test, *G* = 636.6, df = 5, *P*<0.0001). (b) Proportion of plant–fungal association (*P_ij_*) at the order level. The proportions of associated fungi were significantly different between fagaceous and non-fagaceous plants (*G*-test, *G* = 820.5, df = 17, *P*<0.0001). (c) Proportion of plant–fungal association (*P_ij_*) at the genus level. The proportions of associated fungi were significantly different between fagaceous and non-fagaceous plants (*G*-test, *G* = 669.5, df = 18, *P*<0.0001). (d) Proportion of plant–fungal association (*P_ij_*) in terms of fungal functional group. EcM, ectomycorrhizal fungi; AM, arbuscular mycorrhizal fungi; Unknown, fungi with unknown ecological functions. The proportions of associated fungi were significantly different between fagaceous and non-fagaceous plants (*G*-test, *G* = 866.5, df = 2, *P*<0.0001). (e) CLAM analysis of fungi on fagaceous and non-fagaceous plants. Fungal OTUs were classified into the following categories: fungi preferentially associated with fagaceous plants (diamond), fungi preferentially associated with non-fagaceous plants (square), fungi commonly associated with both fagaceous and non-fagaceous plants (circle), and fungi that were rare in both types of host plants (triangle). The OTUs classified in the CLAM analysis are shown in Data S3.
(PDF)

Figure S3 Number of fungal OTUs per sample based on two different data treatment methods. (a) Mean ± SD of the number of OTUs per sample based on a rarefaction approach. In the rarefaction process, the number of sequencing reads per sample was rarefied to 50; hence, samples with less than 50 reads were excluded. After the rarefaction process, a matrix representing the presence/absence of each fungal OTU in each root sample was constructed (Data S3). Data are shown for plant species with 10 or more root samples. (b) Mean ± SD of the number of OTUs per sample based on the 5% cutoff method. In the 5%-cutoff data treatment process, only OTUs with more than 5% of the sample total reads were designated as present in a sample (Data S3). Note that the mean number of fungal OTUs per sample was smaller for fagaceous plant species (shaded) than for non-fagaceous plant species regardless of data treatment methods. The observed lower diversity of fungal OTUs in fagaceous root samples may be attributed to the formation of dense mycelial mat (i.e., “mantle”) by ectomycorrhizal fungi, whose mycelia often envelope the root tips of fagaceous plants [2].
(PDF)

Figure S4 Community-ecological analysis based on rarefied dataset (CLAM analysis of fungi on fagaceous and non-fagaceous plants). To examine the robustness of the community-ecological analysis in terms of the two alternative data treatment methods detailed in Figure S3, the CLAM analysis (Fig. 2b) was re-conducted based on the rarefied dataset (Fig. S3b; Data S4). Fungal OTUs were classified into the following categories: fungi preferentially associated with fagaceous plants (diamond), fungi preferentially associated with non-fagaceous plants (square), fungi commonly associated with both fagaceous and non-fagaceous plants (circle), and fungi that were rare in both types of host plants (triangle). See Data S3 for the detailed results of the classification analysis.
(PDF)

Figure S5 Community-ecological analysis based on rarefied dataset (number of ectomycorrhizal and arbuscular mycorrhizal fungal OTUs shared among plant species). To examine the robustness of the community-ecological analysis in terms of the two alternative data treatment methods detailed in Figure S3, the number of ectomycorrhizal and arbuscular mycorrhizal fungal OTUs shared among plant species (Fig. 3) was re-calculated based on the rarefied dataset (Fig. S3b; Data S5). (a) Number of shared ectomycorrhizal fungal OTUs. The line thickness is proportional to the number of fungal OTUs shared in each pair of plant species. The size of circles roughly represents the relative abundance of plant species that was evaluated by the number of root samples. (b) Number of shared arbuscular mycorrhizal OTUs.
(PDF)

Figure S6 Fungal OTU composition. (a) Phylum-level composition of fungal OTUs observed in root samples. The numbers shown in the graph indicate the number of OTUs belonging to respective taxa. (b) Order-level composition of fungal OTUs. (c) Genus-level composition of fungal OTUs. (d) Number of ectomycorrhizal and arbuscular mycorrhizal fungal OTUs. The number of fungal OTUs with unknown ecological functions is also shown.
(PDF)

Figure S7 Number of fungal OTUs shared among plant species (all fungal OTUs). The line thickness is proportional to the number of fungal OTUs shared between each pair of plant species. The size of circles roughly represents the relative abundance of plant species that was evaluated by the number of root samples (Fig. S1c).
(PDF)

Figure S8 Mantel’s correlogram analysis applied separately for fagaceous-plant and non-fagaceous-plant root samples. (a) Spatial autocorrelation of root-associated fungal OTU composition on fagaceous plants. At each distance class, Mantel’s correlation between spatial distance and dissimilarity of root-associated fungal composition (Raup-Crick *β*-diversity) was examined. Filled squares represent statistically significant correlation (*P*<0.05) after Bonferroni correction. (b) Spatial autocorrelation of root-associated fungal OTU composition on non-fagaceous plants.
(PDF)

Data S1 Fungal OTU sequences in FASTA format (95% cutoff similarity).
(TXT)

Data S2 Fungal OTU sequences in FASTA format (97% cutoff similarity).
(TXT)

Data S3 Fungal OTUs detected from the root samples.
(XLSX)

Data S4 Matrix representing the presence/absence of fungal OTUs in each root sample.
(XLSX)

Data S5 Matrix representing the symbiosis of plant species and fungal OTUs.
(XLSX)

Data S6 Matrices used for the *G*-tests comparing the proportion of associated fungi between fagaceous and non-fagaceous plants.
(XLSX)

## References

[1] Brundrett MC (2009) Mycorrhizal associations and other means of nutrition of vascular plants: understanding the global diversity of host plants by resolving conflicting information and developing reliable means of diagnosis. Plant Soil 320: 37–77.

[2] Smith SE, Read DJ (2008) Mycorrhizal symbiosis, 3rd edition. New York: Elsevier.

[3] Fellbaum CR, Gachomo EW, Beesetty Y, Choudhari S, Strahan GD, et al (2012) Carbon availability triggers fungal nitrogen uptake and transport in arbuscular mycorrhizal symbiosis. Proc Natl Acad Sci, USA 109: 2666–2671. 22308426 10.1073/pnas.1118650109 PMC3289346

[4] Nara K (2006) Ectomycorrhizal networks and seedling establishment during early primary succession. New Phytol 169: 169–178. 16390428 10.1111/j.1469-8137.2005.01545.x

[5] Smith SE, Smith FA (2011) Roles of arbuscular mycorrhizas in plant nutrition and growth: new paradigms from cellular to ecosystem scales. Ann Rev Plant Biol 62: 227–250. 21391813 10.1146/annurev-arplant-042110-103846

[6] Finlay R, Read D (1986) The structure and function of the vegetative mycelium of ectomycorrhizal plants. II. The uptake and distribution of phosphorus by mycelial strands interconnecting host plants. New Phytol 103: 157–165.

[7] Wu B, Maruyama H, Teramoto M, Hogetsu T (2012) Structural and functional interactions between extraradical mycelia of ectomycorrhizal *Pisolithus* isolates. New Phytol 194: 1070–1078. 22471555 10.1111/j.1469-8137.2012.04126.x

[8] Kiers ET, Duhamel M, Beesetty Y, Mensah JA, Franken O, et al (2011) Reciprocal rewards stabilize cooperation in the mycorrhizal symbiosis. Science 333: 880–882. 21836016 10.1126/science.1208473

[9] Högberg MN, Högberg P (2002) Extramatrical ectomycorrhizal mycelium contributes one-third of microbial biomass and produces, together with associated roots, half the dissolved organic carbon in a forest soil. New Phytol 154: 791–795. 10.1046/j.1469-8137.2002.00417.x 33873454

[10] Högberg P, Nordgren A, Buchmann N, Taylor AFS, Ekblad A, et al (2001) Large-scale forest girdling shows that current photosynthesis drives soil respiration. Nature 411: 789–792. 11459055 10.1038/35081058

[11] Phillips RP, Brzostek E, Midgley MG (2013) The mycorrhizal-associated nutrient economy: a new framework for predicting carbon–nutrient couplings in temperate forests. New Phytol 199: 41–51. 23713553 10.1111/nph.12221

[12] Read DJ, Perez-Moreno J (2003) Mycorrhizas and nutrient cycling in ecosystems-a journey towards relevance? New Phytol 157: 475–492. 10.1046/j.1469-8137.2003.00704.x 33873410

[13] van der Heijden MG, Klironomos JN, Ursic M, Moutoglis P, Streitwolf-Engel R, et al (1998) Mycorrhizal fungal diversity determines plant biodiversity, ecosystem variability and productivity. Nature 396: 69–72.

[14] Verbruggen E, van der Heijden MGA, Rillig MC, Kiers ET (2013) Mycorrhizal fungal establishment in agricultural soils: factors determining inoculation success. New Phytol 197: 1104–1109. 23495389 10.1111/j.1469-8137.2012.04348.x

[15] Redecker D, Kodner R, Graham LE (2000) Glomalean fungi from the Ordovician. Science 289: 1920–1921. 10988069 10.1126/science.289.5486.1920

[16] Bever JD, Schultz PA, Pringle A, Morton JB (2001) Arbuscular mycorrhizal fungi: more diverse than meets the eye, and the ecological tale of why. Bioscience 51: 923–932.

[17] Tedersoo L, May TW, Smith ME (2010) Ectomycorrhizal lifestyle in fungi: global diversity, distribution, and evolution of phylogenetic lineages. Mycorrhiza 20: 217–263. 20191371 10.1007/s00572-009-0274-x

[18] Peay KG, Kennedy PG, Davies SJ, Tan S, Bruns TD (2010) Potential link between plant and fungal distributions in a dipterocarp rainforest: community and phylogenetic structure of tropical ectomycorrhizal fungi across a plant and soil ecotone. New Phytol 185: 529–542. 19878464 10.1111/j.1469-8137.2009.03075.x

[19] Öpik M, Vanatoa A, Vanatoa E, Moora M, Davison J, et al (2010) The online database MaarjAM reveals global and ecosystemic distribution patterns in arbuscular mycorrhizal fungi (Glomeromycota). New Phytol 188: 223–241. 20561207 10.1111/j.1469-8137.2010.03334.x

[20] Öpik M, Zobel M, Cantero JJ, Davison J, Facelli JM, et al (2013) Global sampling of plant roots expands the described molecular diversity of arbuscular mycorrhizal fungi. Mycorrhiza 23: 1–20. 23422950 10.1007/s00572-013-0482-2

[21] Tedersoo L, Bahram M, Toots M, DiÉDhiou AG, Henkel TW, et al (2012) Towards global patterns in the diversity and community structure of ectomycorrhizal fungi. Mol Ecol 21: 4160–4170. 22568722 10.1111/j.1365-294X.2012.05602.x

[22] Lindahl BD, Nilsson RH, Tedersoo L, Abarenkov K, Carlsen T, et al (2013) Fungal community analysis by high-throughput sequencing of amplified markers – a user’s guide. New Phytol 199: 288–299. 23534863 10.1111/nph.12243 PMC3712477

[23] Nilsson HR, Tedersoo L, Lindahl BD, Kjøller R, Carlsen T, et al (2011) Towards standardization of the description and publication of next-generation sequencing datasets of fungal communities. New Phytol 191: 314–318. 21557749 10.1111/j.1469-8137.2011.03755.x

[24] Jumpponen A, Jones KL, David MJ, Yaege C (2010) Massively parallel 454-sequencing of fungal communities in *Quercus* spp. ectomycorrhizas indicates seasonal dynamics in urban and rural sites. Mol Ecol 19: 41–53. 20331769 10.1111/j.1365-294X.2009.04483.x

[25] Öpík M, Metsis M, Daniell TJ, Zobel M, Moora M (2009) Large-scale parallel 454 sequencing reveals host ecological group specificity of arbuscular mycorrhizal fungi in a boreonemoral forest. New Phytol 184: 424–437. 19558424 10.1111/j.1469-8137.2009.02920.x

[26] Toju H, Yamamoto S, Sato H, Tanabe AS (2013) Sharing of diverse mycorrhizal and root-endophytic fungi among plant species in an oak-dominated cool-temperate forest. PLoS ONE 8: e78248. 24250752 10.1371/journal.pone.0078248 PMC3824041

[27] McGuire K, Henkel T, de la Cerda IG, Villa G, Edmund F, et al (2008) Dual mycorrhizal colonization of forest-dominating tropical trees and the mycorrhizal status of non-dominant tree and liana species. Mycorrhiza 18: 217–222. 18365256 10.1007/s00572-008-0170-9

[28] Tedersoo L, Nilsson RH, Abarenkov K, Jairus T, Sadam A, et al (2010) 454 Pyrosequencing and Sanger sequencing of tropical mycorrhizal fungi provide similar results but reveal substantial methodological biases. New Phytol 188: 291–301. 20636324 10.1111/j.1469-8137.2010.03373.x

[29] Bahram M, Põlme S, Kõljalg U, Tedersoo L (2011) A single European aspen (*Populus tremula*) tree individual may potentially harbour dozens of *Cenococcum geophilum* ITS genotypes and hundreds of species of ectomycorrhizal fungi. FEMS Microbiol Ecol 75: 313–320. 21114502 10.1111/j.1574-6941.2010.01000.x

[30] Husband R, Herre EA, Turner SL, Gallery R, Young JPW (2002) Molecular diversity of arbuscular mycorrhizal fungi and patterns of host association over time and space in a tropical forest. Mol Ecol 11: 2669–2678. 12453249 10.1046/j.1365-294x.2002.01647.x

[31] Zhao Z-W, Xia Y-M, Qin X-Z, Li X-W, Cheng L-Z, et al (2001) Arbuscular mycorrhizal status of plants and the spore density of arbuscular mycorrhizal fungi in the tropical rain forest of Xishuangbanna, southwest China. Mycorrhiza 11: 159–162. 24595436 10.1007/s005720100117

[32] Wu YT, Wubet T, Trogisch S, Both S, Scholten T, et al (2013) Forest age and plant species composition determine the soil fungal community composition in a Chinese subtropical forest. PLoS ONE 8: e66829. 23826151 10.1371/journal.pone.0066829 PMC3694989

[33] Enoki T (2003) Microtopography and distribution of canopy trees in a subtropical evergreen broad-leaved forest in the northern part of Okinawa Island, Japan. Ecol Res 18: 103–113.

[34] Mandyam K, Jumpponen A (2005) Seeking the elusive function of the root-colonising dark septate endophytic fungi. Studies Mycol 53: 173–189.

[35] Toju H, Sato H, Yamamoto S, Kadowaki K, Tanabe AS, et al (2013) How are plant and fungal communities linked to each other in belowground ecosystems? A massively parallel pyrosequencing analysis of the association specificity of root-associated fungi and their host plants. Ecol Evol 3: 3112–3124. 24101998 10.1002/ece3.706 PMC3790555

[36] Toju H, Yamamoto S, Sato H, Tanabe AS, Gilbert GS, et al (2013) Community composition of root-associated fungi in a *Quercus*-dominated temperate forest: “codominance” of mycorrhizal and root-endophytic fungi. Ecol Evol 3: 1281–1293. 23762515 10.1002/ece3.546 PMC3678483

[37] Newsham KK (2011) A meta-analysis of plant responses to dark septate root endophytes. New Phytol 190: 783–793. 21244432 10.1111/j.1469-8137.2010.03611.x

[38] Kageyama S, Mandyam K, Jumpponen A (2008) Diversity, function and potential applications of the root-associated endophytes. In Varma A, ed. Mycorrhiza, 3rd ed. 29–57. Springer, Berlin Heidelberg.

[39] Kennedy P (2010) Ectomycorrhizal fungi and interspecific competition: species interactions, community structure, coexistence mechanisms, and future research directions. New Phytol 187: 895–910. 20673286 10.1111/j.1469-8137.2010.03399.x

[40] Kennedy PG, Bruns TD (2005) Priority effects determine the outcome of ectomycorrhizal competition between two *Rhizopogon* species colonizing Pinus muricata seedlings. New Phytol 166: 631–638. 15819925 10.1111/j.1469-8137.2005.01355.x

[41] Alexander I, Lee S, Burslem D, Pinard M, Hartley S (2005) Mycorrhizas and ecosystem processes in tropical rain forest: implications for diversity. In Burslem DFRP, Pinard MA and Hartley SE eds. Biotic interactions in the tropics: Their role in the maintenance of species diversity. 165–203. Cambridge University Press, Cambridge, UK.

[42] Eguchi T (1984) Climate of Yaku-shima Island, especially regionality of precipitation distribution. In Nature Conservation Bureau, Environment Agency, Japan ed. Conservation Reports of the Yaku-shima Wilderness Area, Kyushu, Japan. 3–26. Nature Conservation Bureau, Environment Agency, Japan, Tokyo. [In Japanese with English abstract].

[43] Montesinos-Navarro A, Segarra-Moragues JG, Valiente-Banuet A, Verdú M (2012) The network structure of plant-arbuscular mycorrhizal fungi. New Phytol 194: 536–547. 22269207 10.1111/j.1469-8137.2011.04045.x

[44] Nielsen A, Bascompte J (2007) Ecological networks, nestedness and sampling effort. J Ecol 95: 1134–1141.

[45] Sato H, Murakami N (2008) Reproductive isolation among cryptic species in the ectomycorrhizal genus *Strobilomyces*: population-level CAPS marker-based genetic analysis. Mol Phyl Evol 48: 326–334. 10.1016/j.ympev.2008.01.033 18331802

[46] Hamady M, Walker JJ, Harris JK, Gold NJ, Knight R (2008) Error-correcting barcoded primers for pyrosequencing hundreds of samples in multiplex. Nature Methods 5: 235–237. 18264105 10.1038/nmeth.1184 PMC3439997

[47] Toju H, Tanabe AS, Yamamoto S, Sato H (2012) High-coverage ITS primers for the DNA-based identification of ascomycetes and basidiomycetes in environmental samples. PLoS ONE 7: e40863. 22808280 10.1371/journal.pone.0040863 PMC3395698

[48] Kunin V, Engelbrektson A, Ochman H, Hugenholtz P (2010) Wrinkles in the rare biosphere: pyrosequencing errors can lead to artificial inflation of diversity estimates. Env Microbiol 12: 118–123. 19725865 10.1111/j.1462-2920.2009.02051.x

[49] Li W, Fu L, Niu B, Wu S, Wooley J (2012) Ultrafast clustering algorithms for metagenomic sequence analysis. Briefings in Bioinformatics 13: 656–668. 22772836 10.1093/bib/bbs035 PMC3504929

[50] Tanabe AS (2013) Assams v0.1.2013.01.01, a software distributed by the author at http://www.fifthdimension.jp/products/assams/.

[51] Sommer DD, Delcher AL, Salzberg SL, Pop M (2007) Minimus: a fast, lightweight genome assembler. BMC Bioinformatics 8: 64. 17324286 10.1186/1471-2105-8-64 PMC1821043

[52] Edgar RC, Haas BJ, Clemente JC, Quince C, Knight R (2011) UCHIME improves sensitivity and speed of chimera detection. Bioinformatics 27: 2194–2200. 21700674 10.1093/bioinformatics/btr381 PMC3150044

[53] Zhang Z, Schwartz S, Wagner L, Miller W (2000) A greedy algorithm for aligning DNA sequences. J Computational Biol 7: 203–214. 10.1089/10665270050081478 10890397

[54] Nilsson RH, Kristiansson E, Ryberg M, Hallenberg N, Larsson KH (2008) Intraspecific ITS variability in the kingdom Fungi as expressed in the international sequence databases and its implications for molecular species identification. Evol Bioinformatics 4: 193–201. 10.4137/ebo.s653 PMC2614188 19204817

[55] Oksanen J, Blanachet FG, Kindt R, Legendre P, Minchin PR, et al. (2012) Vegan: community ecology package. R package version 2.0–3 available at http://CRAN.R-project.org/package=vegan.

[56] Tanabe AS, Toju H (2013) Two new computational methods for universal DNA barcoding: A benchmark using barcode sequences of bacteria, archaea, animals, fungi, and land plants. PLoS ONE 8: e76910. 24204702 10.1371/journal.pone.0076910 PMC3799923

[57] Tanabe AS (2012) Claident v0.1.2012.11.23, a software distributed by author at http://www.fifthdimension.jp/products/claident/.

[58] Camacho C, Coulouris G, Avagyan V, Ma N, Papadopoulos J, et al (2009) BLAST+: architecture and applications. BMC Bioinformatics 10: 421. 20003500 10.1186/1471-2105-10-421 PMC2803857

[59] Huson DH, Auch AF, Qi J, Schuster SC (2007) MEGAN analysis of metagenomic data. Genome Research 17: 377–386. 17255551 10.1101/gr.5969107 PMC1800929

[60] Dickie IA, Koide RT, Fayish AC (2001) Vesicular–arbuscular mycorrhizal infection of *Quercus rubra* seedlings. New Phytol 151: 257–264. 10.1046/j.1469-8137.2001.00148.x 33873380

[61] Wang B, Qiu YL (2006) Phylogenetic distribution and evolution of mycorrhizas in land plants. Mycorrhiza 16: 299–363. 16845554 10.1007/s00572-005-0033-6

[62] Sokal RR, Rohlf FJ (1994) Biometry: the principles and practice of statistics in biological research., 3rd edition. Freeman and Co, New York.

[63] Chazdon RL, Chao A, Colwell RK, Lin SY, Norden N, et al (2011) A novel statistical method for classifying habitat generalists and specialists. Ecology 92: 1332–1343. 21797161 10.1890/10-1345.1

[64] Chase JM, Kraft NJB, Smith KG, Vellend M, Inouye BD (2011) Using null models to disentangle variation in community dissimilarity from variation in *α*-diversity. Ecosphere 2: 19–28.

[65] Comas L, Eissenstat D (2009) Patterns in root trait variation among 25 co-existing North American forest species. New Phytol 182: 919–928. 19383105 10.1111/j.1469-8137.2009.02799.x

[66] Murata H, Yamada A, Maruyama T, Endo N, Yamamoto K, et al (2012) Root endophyte interaction between ectomycorrhizal basidiomycete *Tricholoma matsutake* and arbuscular mycorrhizal tree *Cedrela odorata*, allowing in vitro synthesis of rhizospheric “shiro”. Mycorrhiza 23: 235–242. 23064771 10.1007/s00572-012-0466-7

[67] Chen Y, Brundrett M, Dell B (2000) Effects of ectomycorrhizas and vesicular–arbuscular mycorrhizas, alone or in competition, on root colonization and growth of *Eucalyptus globulus* and *E. urophylla* . New Phytol 146: 545–555.

[68] McHugh TA, Gehring CA (2006) Below-ground interactions with arbuscular mycorrhizal shrubs decrease the performance of pinyon pine and the abundance of its ectomycorrhizas. New Phytol 171: 171–178. 16771992 10.1111/j.1469-8137.2006.01735.x

[69] Caruso T, Rillig MC, Garlaschelli D (2012) On the application of network theory to arbuscular mycorrhizal fungi–plant interactions: the importance of basic assumptions. New Phytol 194: 891–894. 22537105 10.1111/j.1469-8137.2012.04163.x

[70] Selosse MA, Bauer R, Moyersoen B (2002) Basal hymenomycetes belonging to the Sebacinaceae are ectomycorrhizal on temperate deciduous trees. New Phytol 155: 183–195. 10.1046/j.1469-8137.2002.00442.x 33873297

[71] Xie X, Huang W, Liu F, Tang N, Liu Y, et al (2013) Functional analysis of the novel mycorrhiza-specific phosphate transporter AsPT1 and PHT1 family from *Astragalus sinicus* during the arbuscular mycorrhizal symbiosis. New Phytol 198: 836–852. 23442117 10.1111/nph.12188

[72] Bonneau L, Huguet S, Wipf D, Pauly N, Truong HN (2013) Combined phosphate and nitrogen limitation generates a nutrient stress transcriptome favorable for arbuscular mycorrhizal symbiosis in *Medicago truncatula* . New Phytol 199: 188–202. 23506613 10.1111/nph.12234

[73] Tisserant E, Da Silva C, Kohler A, Morin E, Wincker P, et al (2011) Deep RNA sequencing improved the structural annotation of the *Tuber melanosporum* transcriptome. New Phytol 189: 883–891. 21223284 10.1111/j.1469-8137.2010.03597.x

