# Supplementary figures and images for: Diversity and Spatial Structure of Belowground Plant–Fungal Symbiosis in a Mixed Subtropical Forest of Ectomycorrhizal and Arbuscular Mycorrhizal Plants
Source: PLoS One. 2014 Jan 28;9(1):e86566. doi: 10.1371/journal.pone.0086566 (PMC3904951; doi:10.1371/journal.pone.0086566)

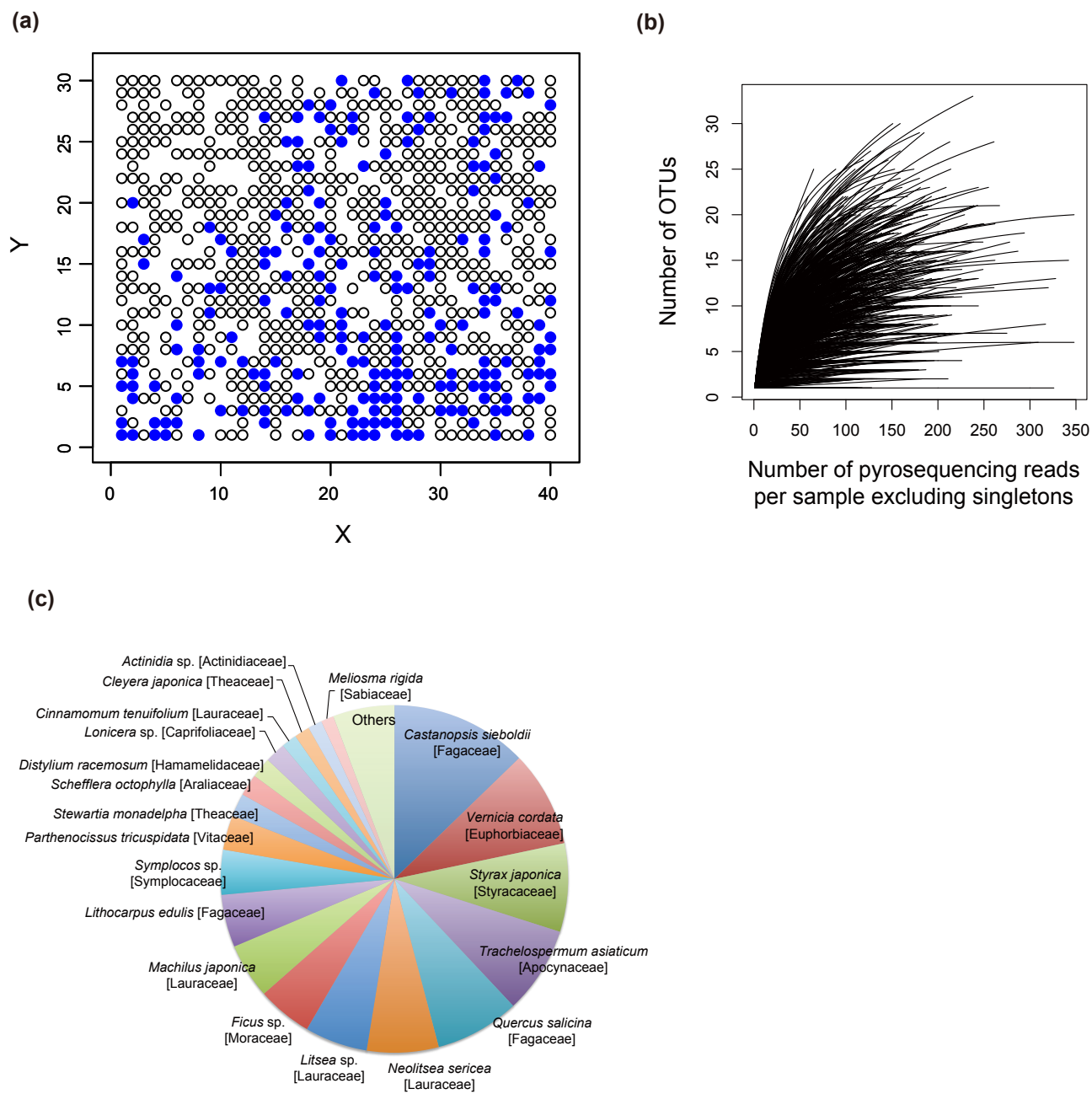

Fig. S1

Supplement: Figure S1 — Sampling locations and the summary of the pyrosequencing. (a) Distribution of fagaceous and non-fagaceous root samples at the study plot. Root samples were collected at 1-m intervals. Filled and open circles represent fagaceous and non-fagaceous root samples, respectively. See Data S3 for detailed information of each root sample. (b) Rarefaction curve of the number of OTUs in each root sample against the number of pyrosequencing reads excluding singletons. (c) Composition of host plant species identified by chloroplast rbcL sequences. The proportion of each plant species among the 849 root samples is shown. (PDF) [file pone.0086566.s001.pdf]

(a) Phylum

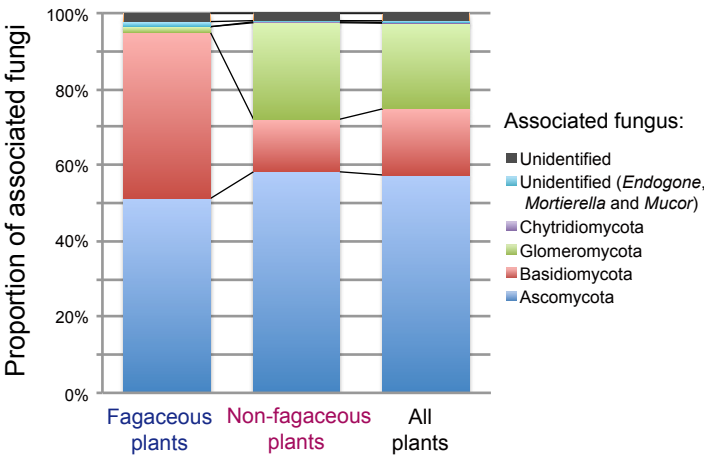

(d) Functional group

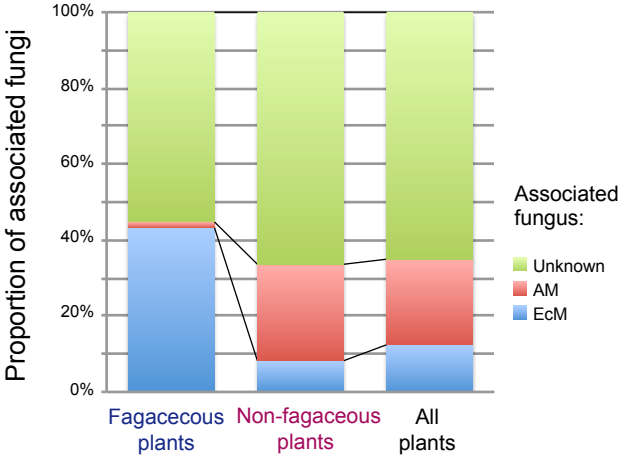

(b) Order

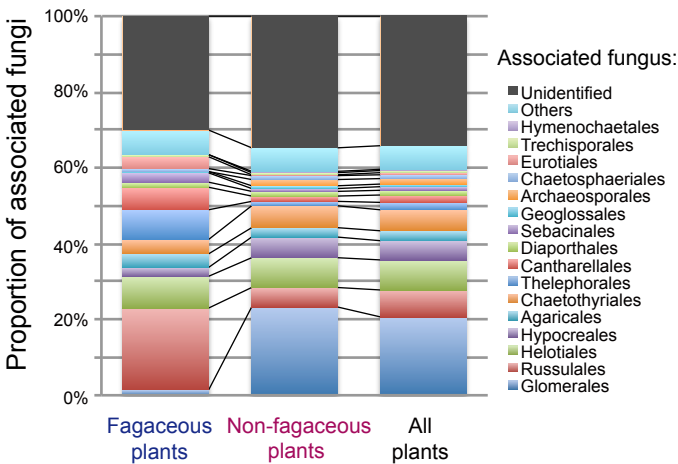

(e) CLAM test

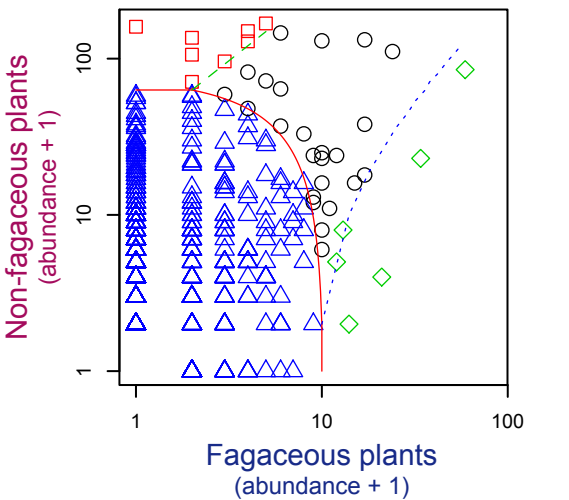

(c) Genus

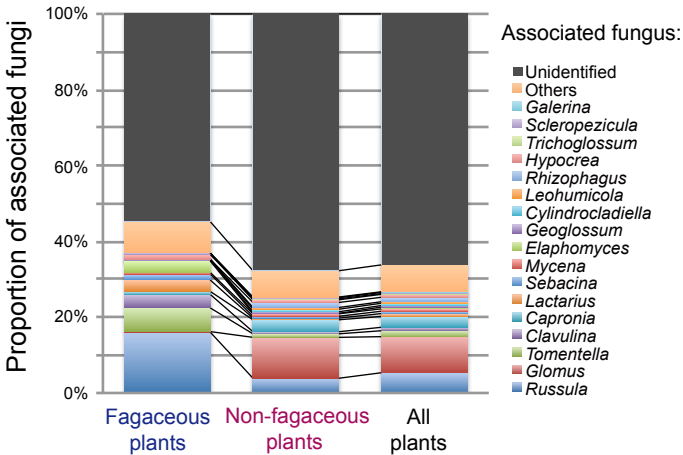

Fig. S2

Supplement: Figure S2 — Results of community ecological analyses based on 97% cutoff similarity for the assembling of fungal OTUs. Additional statistical analyses were conducted based on the 97% cutoff similarity setting of ITS sequences. The exclusion of the OTUs that represented 5% or less of sample total reads (see text) was not applied in this additional analysis to confirm the robustness of the downstream statistical results to alternative data treatments. Compare the results on 97% cutoff similarity (this supplementary figure) with those on 95% cutoff similarity (Figs. 1 and 2). (a) Proportion of plant–fungal association (Pij) at the phylum level. Results are shown for fagaceous (214 root samples), non-fagaceous (635 root samples), and all (fagaceous+non-fagaceous; 214+635 = 849 root samples) plant species. In total, 895, 6562, and 7457 plant–fungal associations were observed for fagaceous, non-fagaceous, and all plant species, respectively. The proportions of associated fungi were significantly different between fagaceous and non-fagaceous plants (G-test, G = 636.6, df = 5, P<0.0001). (b) Proportion of plant–fungal association (Pij) at the order level. The proportions of associated fungi were significantly different between fagaceous and non-fagaceous plants (G-test, G = 820.5, df = 17, P<0.0001). (c) Proportion of plant–fungal association (Pij) at the genus level. The proportions of associated fungi were significantly different between fagaceous and non-fagaceous plants (G-test, G = 669.5, df = 18, P<0.0001). (d) Proportion of plant–fungal association (Pij) in terms of fungal functional group. EcM, ectomycorrhizal fungi; AM, arbuscular mycorrhizal fungi; Unknown, fungi with unknown ecological functions. The proportions of associated fungi were significantly different between fagaceous and non-fagaceous plants (G-test, G = 866.5, df = 2, P<0.0001). (e) CLAM analysis of fungi on fagaceous and non-fagaceous plants. Fungal OTUs were classified into the following categories: fungi prefe [file pone.0086566.s002.pdf]

(a) 5% cutoff

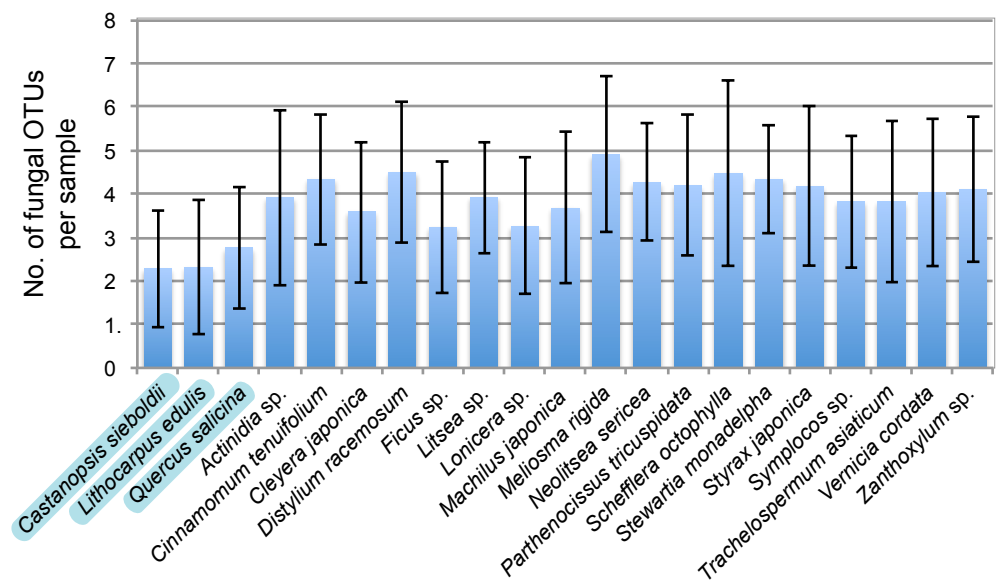

(b) Rarefaction

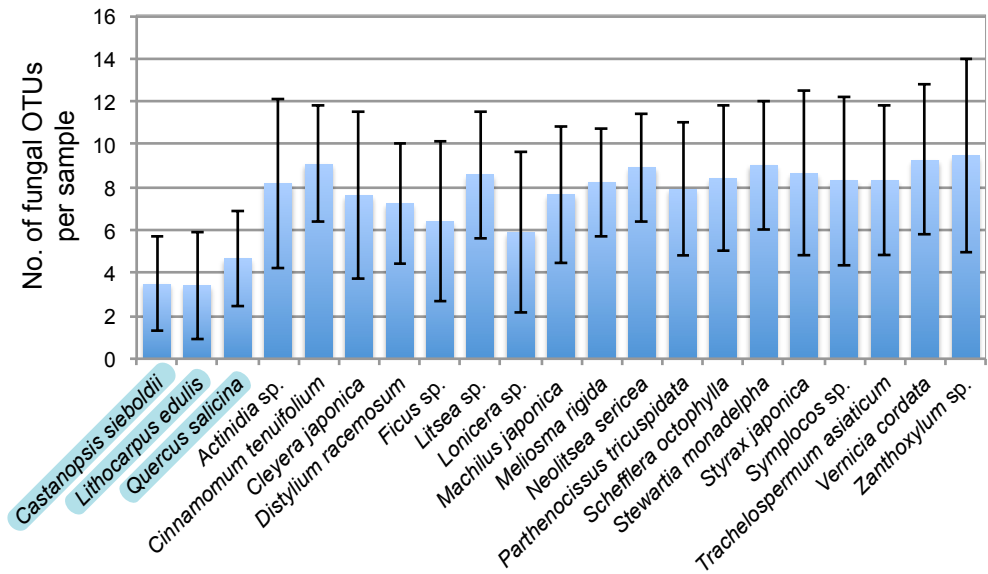

Fig. S3

Supplement: Figure S3 — Number of fungal OTUs per sample based on two different data treatment methods. (a) Mean ± SD of the number of OTUs per sample based on a rarefaction approach. In the rarefaction process, the number of sequencing reads per sample was rarefied to 50; hence, samples with less than 50 reads were excluded. After the rarefaction process, a matrix representing the presence/absence of each fungal OTU in each root sample was constructed (Data S3). Data are shown for plant species with 10 or more root samples. (b) Mean ± SD of the number of OTUs per sample based on the 5% cutoff method. In the 5%-cutoff data treatment process, only OTUs with more than 5% of the sample total reads were designated as present in a sample (Data S3). Note that the mean number of fungal OTUs per sample was smaller for fagaceous plant species (shaded) than for non-fagaceous plant species regardless of data treatment methods. The observed lower diversity of fungal OTUs in fagaceous root samples may be attributed to the formation of dense mycelial mat (i.e., “mantle”) by ectomycorrhizal fungi, whose mycelia often envelope the root tips of fagaceous plants [2]. (PDF) [file pone.0086566.s003.pdf]

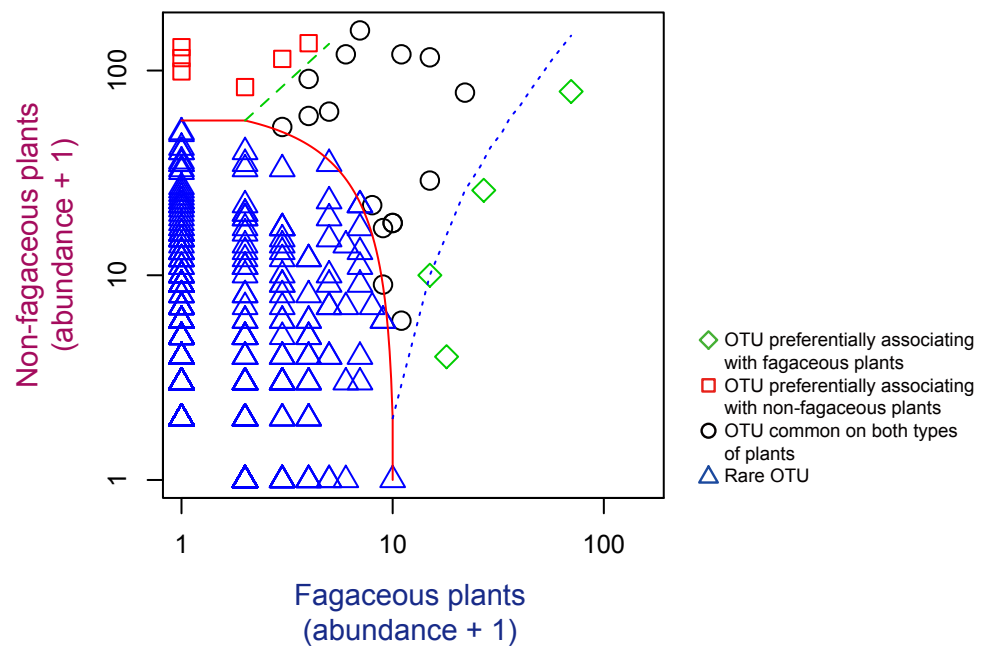

Fig. S4

Supplement: Figure S4 — Community-ecological analysis based on rarefied dataset (CLAM analysis of fungi on fagaceous and non-fagaceous plants). To examine the robustness of the community-ecological analysis in terms of the two alternative data treatment methods detailed in Figure S3, the CLAM analysis (Fig. 2b) was re-conducted based on the rarefied dataset (Fig. S3b; Data S4). Fungal OTUs were classified into the following categories: fungi preferentially associated with fagaceous plants (diamond), fungi preferentially associated with non-fagaceous plants (square), fungi commonly associated with both fagaceous and non-fagaceous plants (circle), and fungi that were rare in both types of host plants (triangle). See Data S3 for the detailed results of the classification analysis. (PDF) [file pone.0086566.s004.pdf]

(a) Ectomycorrhizal  
fungi

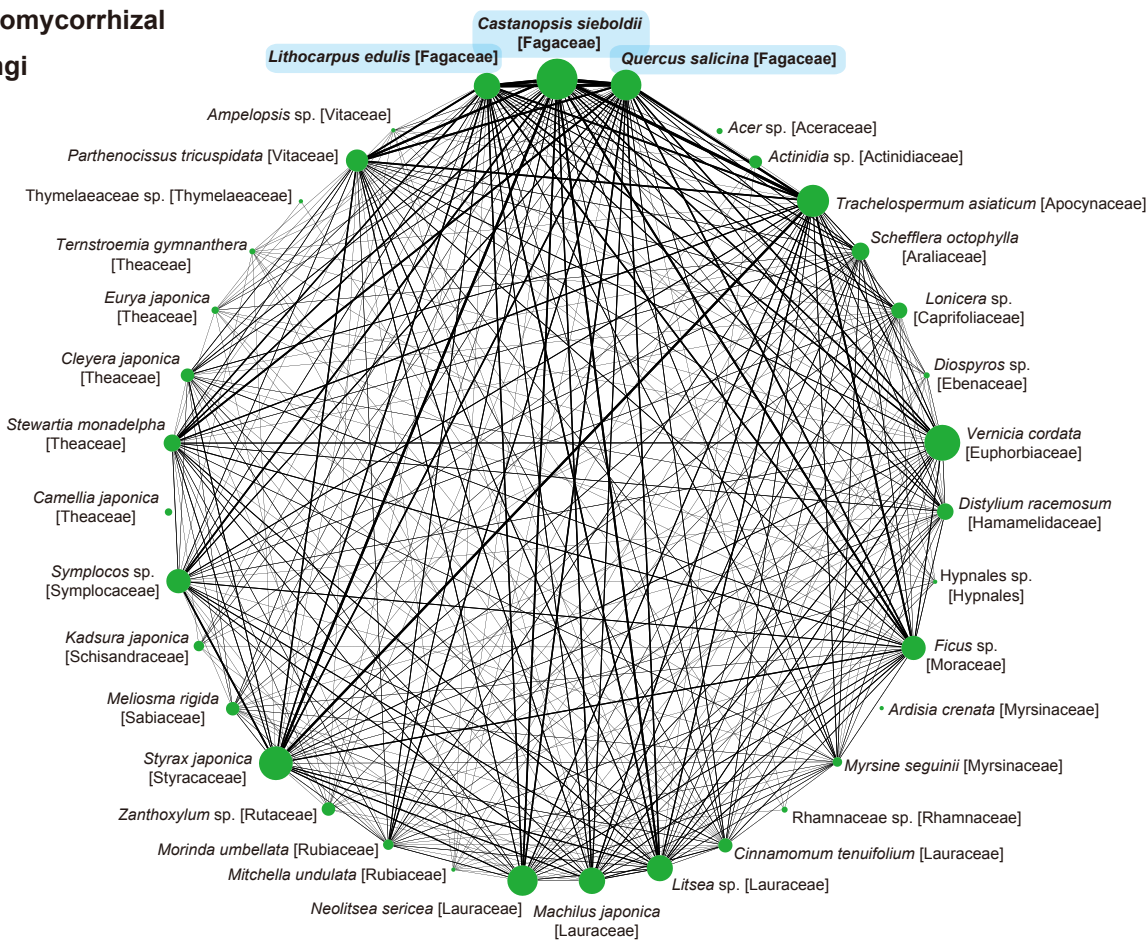

(b) Arbuscular-mycorrhizal  
fungi

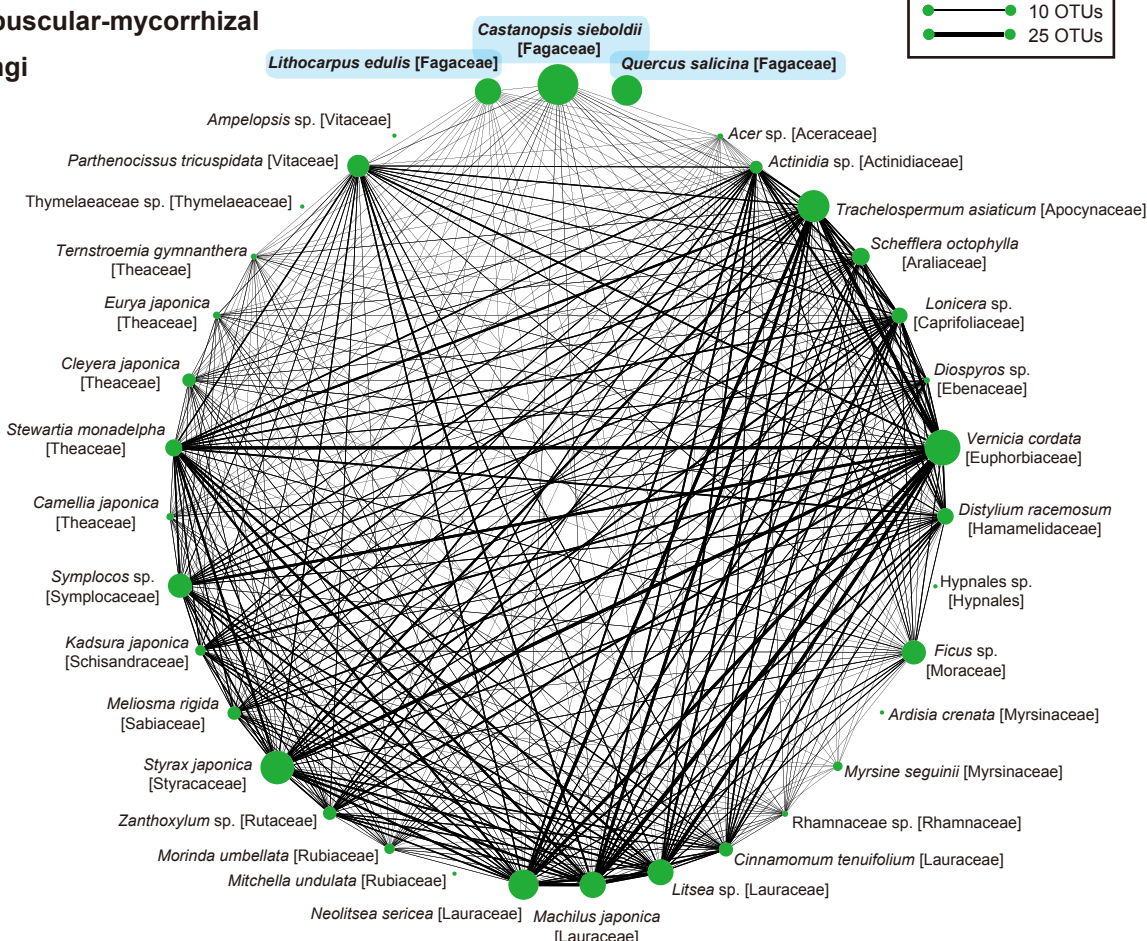

Fig. S5

Supplement: Figure S5 — Community-ecological analysis based on rarefied dataset (number of ectomycorrhizal and arbuscular mycorrhizal fungal OTUs shared among plant species). To examine the robustness of the community-ecological analysis in terms of the two alternative data treatment methods detailed in Figure S3, the number of ectomycorrhizal and arbuscular mycorrhizal fungal OTUs shared among plant species (Fig. 3) was re-calculated based on the rarefied dataset (Fig. S3b; Data S5). (a) Number of shared ectomycorrhizal fungal OTUs. The line thickness is proportional to the number of fungal OTUs shared in each pair of plant species. The size of circles roughly represents the relative abundance of plant species that was evaluated by the number of root samples. (b) Number of shared arbuscular mycorrhizal OTUs. (PDF) [file pone.0086566.s005.pdf]

**(a) Phylum**

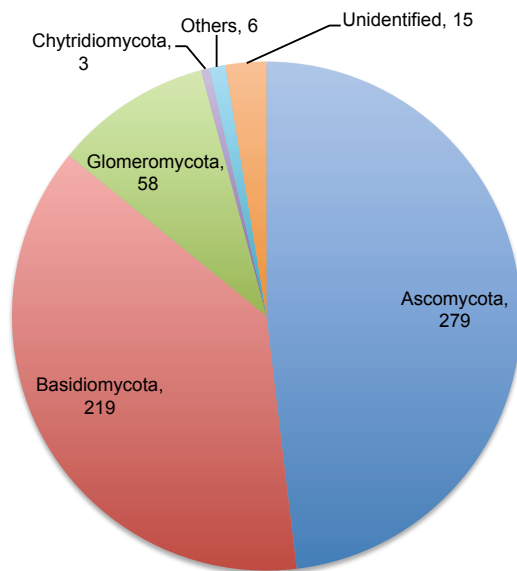

**(b) Order**

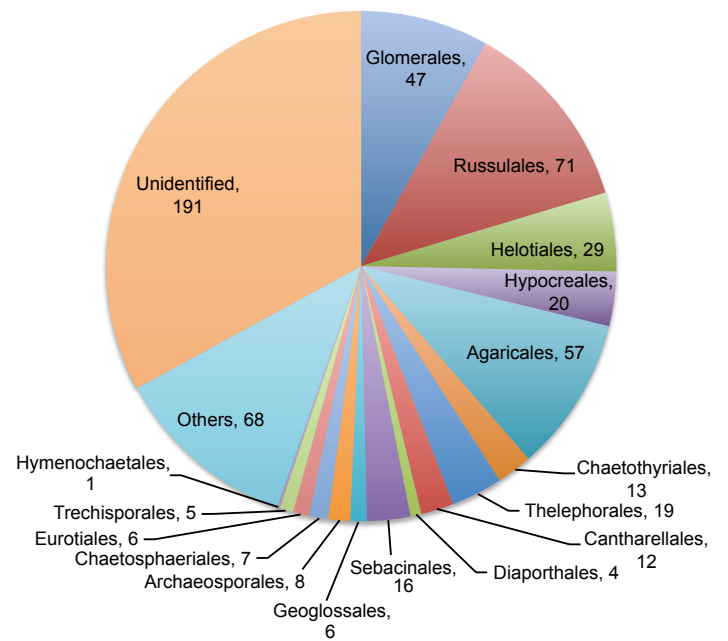

**(c) Genus**

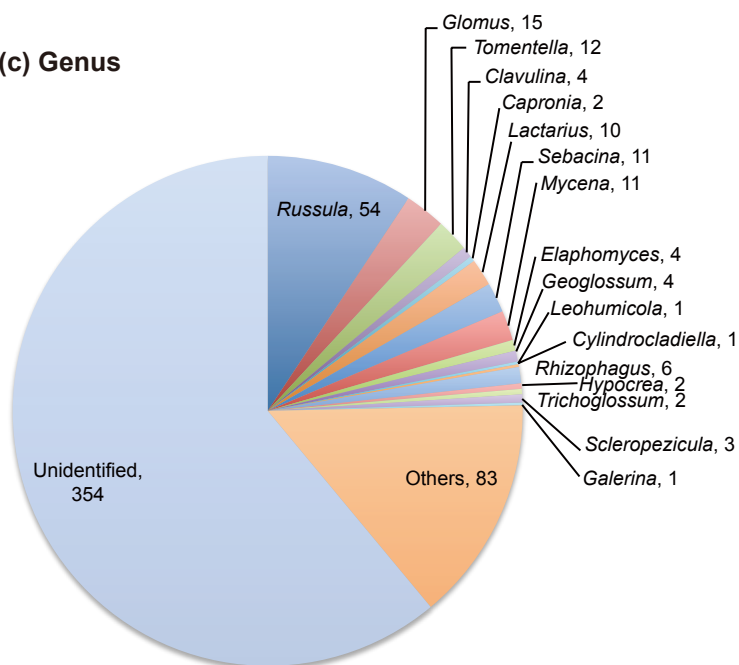

**(d) Functional group**

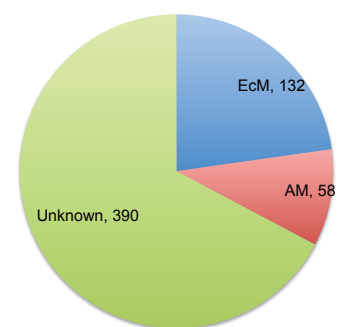

**Fig. S6**

Supplement: Figure S6 — Fungal OTU composition. (a) Phylum-level composition of fungal OTUs observed in root samples. The numbers shown in the graph indicate the number of OTUs belonging to respective taxa. (b) Order-level composition of fungal OTUs. (c) Genus-level composition of fungal OTUs. (d) Number of ectomycorrhizal and arbuscular mycorrhizal fungal OTUs. The number of fungal OTUs with unknown ecological functions is also shown. (PDF) [file pone.0086566.s006.pdf]

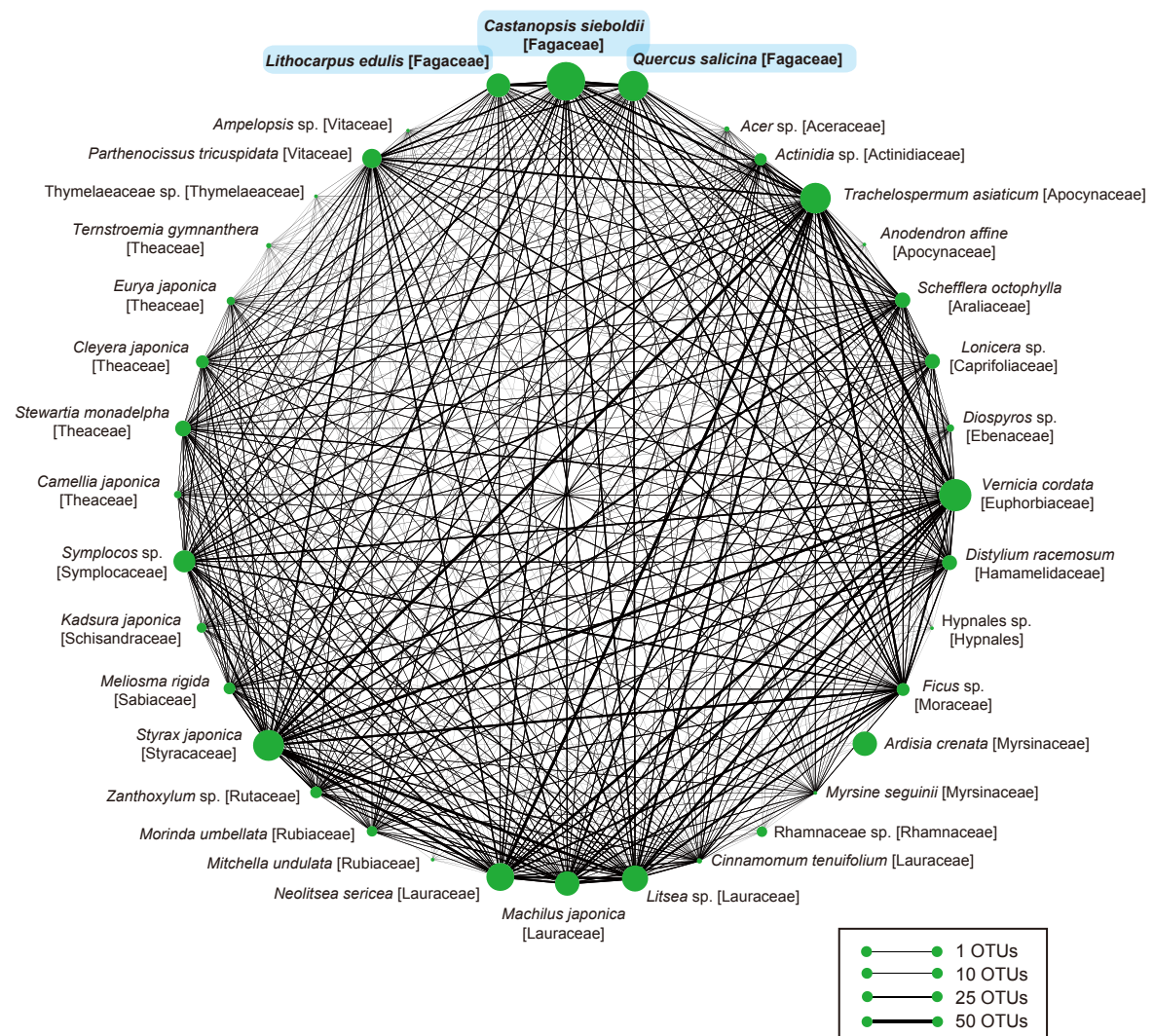

Fig. S7

Supplement: Figure S7 — Number of fungal OTUs shared among plant species (all fungal OTUs). The line thickness is proportional to the number of fungal OTUs shared between each pair of plant species. The size of circles roughly represents the relative abundance of plant species that was evaluated by the number of root samples (Fig. S1c). (PDF) [file pone.0086566.s007.pdf]

(a) Fagaceous plant roots

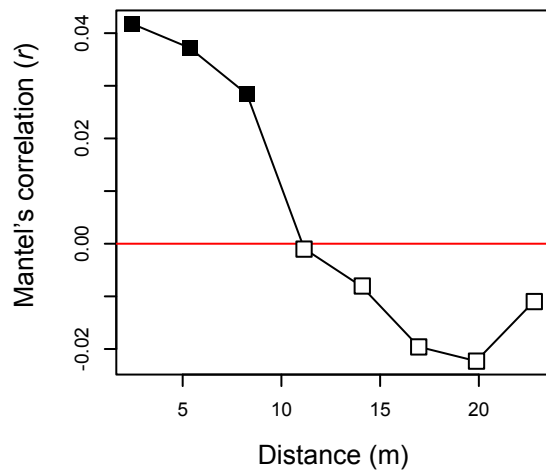

(b) Non-fagaceous plant roots

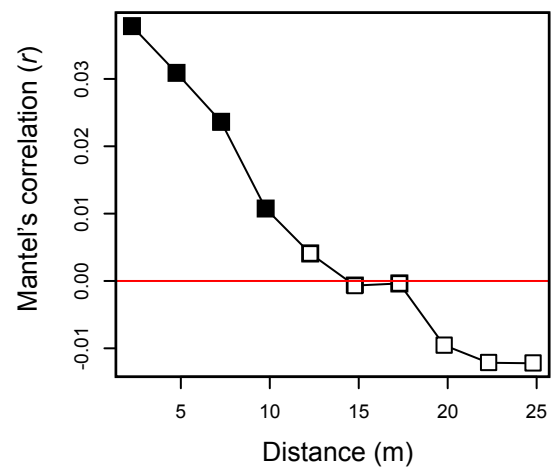

Fig. S8

Supplement: Figure S8 — Mantel’s correlogram analysis applied separately for fagaceous-plant and non-fagaceous-plant root samples. (a) Spatial autocorrelation of root-associated fungal OTU composition on fagaceous plants. At each distance class, Mantel’s correlation between spatial distance and dissimilarity of root-associated fungal composition (Raup-Crick β-diversity) was examined. Filled squares represent statistically significant correlation (P<0.05) after Bonferroni correction. (b) Spatial autocorrelation of root-associated fungal OTU composition on non-fagaceous plants. (PDF) [file pone.0086566.s008.pdf]
